# Bacterial community composition and function in different habitats in Antarctic Fildes region revealed by high-throughput sequencing

**DOI:** 10.3389/fmicb.2025.1524681

**Published:** 2025-06-16

**Authors:** Yi-He Zhang, Yong-Qiang Hu, Yin-Xin Zeng, Ting Hu, Wei Han, Yu Du, Zhong Hu, Shan-Shan Meng

**Affiliations:** ^1^College of Science, Shantou University, Shantou, China; ^2^Key Laboratory for Polar Science, Polar Research Institute of China, Ministry of Natural Resources, Shanghai, China; ^3^School of Oceanography, Shanghai Jiao Tong University, Shanghai, China; ^4^Shanghai Key Laboratory of Polar Life and Environment Sciences, Shanghai Jiao Tong University, Shanghai, China; ^5^Key Laboratory of Polar Ecosystem and Climate Change, Shanghai Jiao Tong University, Ministry of Education, Shanghai, China; ^6^Antarctic Great Wall Ecology National Observation and Research Station, Polar Research Institute of China, Ministry of Natural Resources, Shanghai, China

**Keywords:** bacterial community, marine habitat, terrestrial habitat, Antarctic, metagenomics

## Abstract

**Introduction:**

Pristine soil, ornithogenic soil, intertidal sediment, and marine sediment represent four of typical habitats in the Fildes region, maritime Antarctica. However, information on bacterial community composition and function in these Antarctic habitats remain limited.

**Methods:**

In this study, using a combination of 16S rRNA gene amplicon sequencing and shotgun metagenomic sequencing, 12 samples collected from various habitats in the region were analyzed.

**Results and discussion:**

Bacterial community compositions in terrestrial habitats (i.e., pristine and ornithogenic soils) were found to be distinct (*p* < 0.01) from those in marine habitats (i.e., marine and intertidal sediments). Organic carbon (*p* < 0.01) and pH (*p* < 0.01) were two major environmental factors affecting the bacterial community composition in the diverse habitats. *Proteobacteria* (represented by *Gamma*-, *Alpha*-, and *Betaproteobacteria*; > 30%), *Actinobacteria* (represented by *Actinobacteria*; > 20%), and *Bacteroidetes* (represented by *Flavobacteriia*; > 10%) were dominant in bacteria related to carbon, nitrogen, and sulfur metabolism across all samples. Though most metabolic pathways were common in both terrestrial and marine habitats, terrestrial samples showed more diverse metabolic pathways than marine samples. However, among the top 15 abundant metabolic pathways, genes related to 11 metabolic pathways were relatively more abundant in marine habitats than in terrestrial habitats (*p* < 0.05). More abundant genes related to methane metabolism (e.g., *pmoA*), nitrification (e.g., *amoA* and *hao*), reductive citrate cycle pathway (e.g., *frdA*), repair of DNA damage (e.g., *lexA* and *uvrB*), salt and osmotic stress tolerance (e.g., *betB*, *gltB*, and *treS*), and aromatic hydrocarbon degradation (e.g., *bcrC* and *bssA*) were detected in pristine and/or ornithogenic soils, whereas genes related to sulfur metabolism (e.g., *soxY*, *fccB*, *dsrAB*, and *sat*), nitrogen fixation (e.g., *nifH*), acetyl-CoA metabolism (e.g., *acsB*, *cdhD*, and *cdhE*), carbohydrate degradation (e.g., *amyA* and *chiA*), and cold adaptation (e.g., *cspA*, *deaD* and *recQ*) were in higher abundance in marine and/or intertidal sediments. The influence of penguin feces on soil bacterial community composition and ecological function was observed in this study. The study findings will improve our understanding of bacterial community composition and function in various habitats in maritime Antarctica under the background of global climate change.

## 1 Introduction

The maritime Antarctica comprises the western coastal regions and offshore islands of the Antarctic Peninsula (AP) and the Scotia Arc archipelagoes of the South Shetland, South Orkney, and South Sandwich Islands, plus the isolated oceanic islands of Bouvet and Peter I (Varliero et al., 2024). The Fildes region—comprising the Fildes Peninsula, Ardley Island, and smaller islands off the coast—is located in the southwest of King George Island, the South Shetland Islands adjacent to the AP. This region is one of the largest ice-free areas in the maritime Antarctica and characterized by comparatively high biodiversity due to its slightly higher summer temperatures and greater precipitation than in the continental Antarctica.^1^ Besides vascular plants, mosses, lichens, and terrestrial algae are abundant in the Fildes region. Seabirds (e.g., penguins, skuas, and petrels) and seals are common in coastal areas, exerting a significant impact on soil development via allochthonous organic matter supply (Lupachev et al., 2020). Microorganisms (e.g., bacteria, archaea, fungi, and algae) dominate biomass, biodiversity, and metabolic activity in Antarctic ecosystems (Pearce, 2008; Varliero et al., 2024). These microorganisms are not only sensitive to external disturbances, such as climate warming or human impacts (Bargagli, 2005), but are also important contributors to global climatic and biogeochemical cycles (Hughes et al., 2015; Franco et al., 2017; Cao et al., 2020) and represent simplified global sentinels providing insights into how biodiversity will respond to global change (Koerich et al., 2023).

Including the Fildes region, the AP has warmed dramatically in recent decades and are among the most rapidly warming regions on Earth, resulting in the collapse of ice shelves, decrease in permafrost layer and sea ice, retreat of glaciers, and exposure of new terrestrial habitat (Clarke et al., 2007; Roldán et al., 2022). The response of Antarctic microbial communities to climate change has drawn scientists’ attention and become the focus of investigations in the last 2 decades (Yergeau et al., 2007b; Pearce, 2008; Rinnan et al., 2009; Purcell et al., 2023). Terrestrial ice-free regions on the AP are expanding, creating new habitats not only for the succession of microbial communities but also for the colonization of plants and animals (Lee et al., 2017; Silva et al., 2024). High temperatures have had an impact on microbial communities on the AP, including microbial growth rates and productivity (Royles et al., 2013; Purcell et al., 2023), microbial activities, such as methanotrophic activity and humic substances degradation (Kim et al., 2018; Roldán et al., 2022), and fungal species richness and outbreaks (Newsham et al., 2016; Velázquez et al., 2016). Increased temperature may have direct significant effects on soil microbial communities, resulting in better availability of carbon sources for other indigenous microbes and plants, which can have repercussions for long-term elemental cycling and carbon storage (Kim et al., 2018; Purcell et al., 2023). The effect of increased temperature is more significant for bacteria than for fungi (Yergeau et al., 2007a). In marine ecosystems, besides diatoms being replaced by a community of smaller phytoplankton—particularly cryptophytes and *Micromonas* (Grattepanche et al., 2022)—higher phytoplankton biomass and longer blooms are observed in the west AP (Ferreira et al., 2024). This increases the quantity of organic matter transported from surface layers to the ocean floor (Ducklow et al., 2006). Inputs from primary producers of the euphotic zone can contribute to shape the marine sediment microbial communities (Franco et al., 2017). In addition, meltwater of sea ice and glaciers not only modifies seawater salinity, but also delivers nutrients, including different metals (e.g., iron and manganese), into the ocean and underlying sediments, which subsequently influence planktonic (Evans et al., 2017; Grattepanche et al., 2022) and sediment microbial communities (Wunder et al., 2024). Sediment organic matter and abundant bacterial fraction are two of main factors shaping marine sediment communities of the AP (Fonseca et al., 2022).

Pristine soil, ornithogenic soil, intertidal sediment, and marine sediment represent four of typical habitats in the Fildes region, maritime Antarctica. Habitat specialization plays a crucial role in determining microbial community composition, and this is related to deterministic processes driven by contemporary environmental heterogeneity (Wang et al., 2013; Zhang Y. et al., 2018). Bacterial diversity is heterogeneous and dependent on the habitat type (González-Rocha et al., 2017), and bacteria are important bioindicators in different habitats due to their environmental sensitivity (Zhang C. et al., 2024). Therefore, studying bacterial community structure and activity in various habitats in response to temperature fluctuations is critical to understand how Antarctic ecosystems will respond to future warming. Distinct prokaryotic community compositions exist between marine and terrestrial environments due to varying natural conditions. Bacterial phyla *Actinobacteria*, *Acidobacteria*, *Proteobacteria*, *Bacteroidetes*, *Chloroflexi*, *Gemmatimonadetes*, *Firmicutes*, and *Verrucomicrobia*, archaeal phyla *Thaumarchaeota*/*Crenarchaeota* and *Euryarchaeota*, and fungal phyla *Ascomycota*, *Basidiomycota*, *Zygomycota*, and/or *Mortierellomycota* are commonly detected in soils of the Fildes region (Teixeira et al., 2010; Kim et al., 2012; Wang et al., 2015; Ding et al., 2016; Kim et al., 2018; Zhang Y. et al., 2018; Durán et al., 2019; Santos et al., 2020; Nopnakorn et al., 2023). Members of green algae *Trebouxiophyceae* predominate in the soil algae communities (Rybalka et al., 2023). Meanwhile, *Proteobacteria, Bacteroidetes, Firmicutes, Cyanobacteria, Verrucomicrobia*, and *Actinobacteria* are dominant in glacier flows and ice-melt streams (Valdespino-Castillo et al., 2018). In addition, dominant bacteria in lake sediment contain *Proteobacteria, Bacteroidetes, Gemmatimonadetes, Firmicutes*, and *Actinobacteria* (Li et al., 2006). Compared to periglacial lakes dominated by *Bacteroidota, Actinobacteria*, and *Proteobacteria*, marine bacterioplankton communities in coves are dominated by *Proteobacteria* and *Bacteroidota* (Zeng et al., 2014; Zhang C. et al., 2024). In intertidal sediments, *Proteobacteria, Bacteroidetes*, and *Actinobacteria* are the predominant phyla in bacterial communities (Wang et al., 2016), whereas marine sediments have a high abundance of *Proteobacteria*, followed by *Firmicutes, Bacteroidetes*, and *Actinobacteria* (Zhou et al., 2013; Franco et al., 2017). However, compared to terrestrial habitats, data on microbial communities in marine habitats especially in marine sediment in the Fildes region are quite rare.

Despite the importance of microorganisms in driving biogeochemical cycling and fast warming of the AP, information on bacterial community composition and function in various habitats remain limited. Whether there exist obvious differences in community composition as well as ecological function of bacteria inhabiting different habitats is what we concern about. In this study, amplicon sequencing and metagenomic sequencing were utilized to study bacterial community structure and function in different habitats in the Fildes region. The impact of different environmental factors on bacterial communities was further analyzed. In addition, functional differences in carbon, nitrogen, and sulfur cycling in different habitats were analyzed and compared using metagenomic method. Knowledge of bacterial composition, indicator species, and their functional potential is not only vital for an improved understanding of the role of bacteria in terrestrial and oceanic ecosystem processes in the maritime Antarctic region, but is also helpful to predict the response of bacterial communities to environmental conditions under the background of global climate change.

## 2 Materials and methods

### 2.1 Sample collection

**FIGURE 1:**
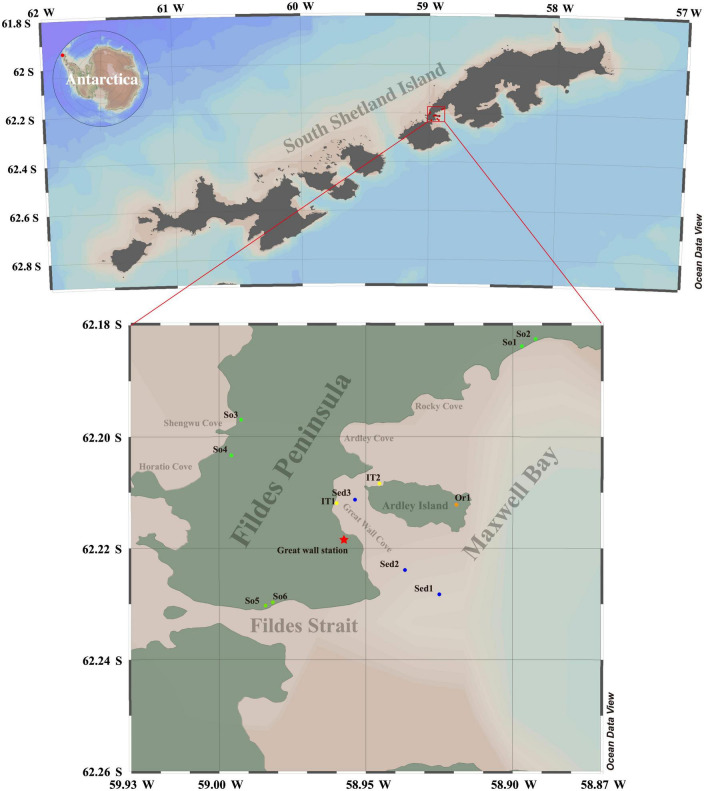
Map of sampling sites in Antarctic Fildes region. So1–So6, pristine soils; Or1, ornithogenic soil; IT1–IT2, intertidal sediments; Sed1–Sed3, marine sediments.

Twelve samples (approximately 200 g per sample)—representing four typical habitats—were collected from Antarctic Fildes region in January 2018 (Figure 1 and Table 1). Ornithogenic soil sample (Or1) was collected from 0 to 5 cm deep surface layer near the active penguin colony on the eastern part of Ardley Island with a small sterilized shovel. Pristine soil samples (So1, So2, So3, So4, So5, and So6) were collected from 0 to 5 cm deep surface layer at different sites on the Fields Peninsula. Intertidal sediment samples (IT1 and IT2) were collected from 0 to 3 cm deep surface layer at the shore of Great Wall cove and Ardley Island. Marine sediment samples (Sed1, Sed2, and Sed3) were collected from Great Wall cove with a van Veen grab. All samples were stored at –80^°^C until further use. In this study, ocean data view (ODV v5.6.2) was used to produce maps.

**TABLE 1:** Sampling information at different sites.

Sample	Sampling site	Location	Sampling time	Sample type
So1	Close to Artigas station, Near Collins ice cap	62°11′02′′S, 58°53′49′′W	1/11/2018	Pristine soil
So2	Seaside exposed part of Collins ice cap	62°10′58′′S, 58°53′32′′W	1/11/2018	
So3	Biobay, Antarctica	62°11′49′′S, 58°59′33′′W	1/23/2018	
So4	Biobay, Antarctica	62°12′12′′S, 58°59′45′′W	1/23/2018	
So5	Jasper beach, Antarctica	62°13′49′′S, 58°59′03′′W	1/26/2018	
So6	Jasper beach, Antarctica	62°13′47′′S, 58°58′54′′W	1/26/2018	
Or1	Ardley Island, Antarctica	62°12′44′′S, 58°55′09′′W	1/27/2018	Ornithogenic soil
IT1	Near Great Wall station	62°12′43′′S, 58°57′42′′W	1/13/2018	Intertidal sediment
IT2	Penguin island, Antarctica	62°12′35′′S, 58°56′42′′W	1/13/2018	
Sed1	Great Wall cove, Antarctica	62°13′42′′S, 58°55′30′′W	1/16/2018	Marine sediment
Sed2	Great Wall cove, Antarctica	62°13′26′′S, 58°56′12′′W	1/16/2018	
Sed3	Great Wall cove, Antarctica	62°12′40′′S, 58°57′13′′W	1/17/2018	

### 2.2 Geochemical analyses

The pH was measured by adding 25 mL of distilled water to 10 g of sample and recording pH using a benchtop pH meter (Orion Star A211, Thermo Scientific Inc., USA). Ammonium nitrogen (NH_4_**^+^-N**) and nitrate nitrogen (NO_3_**^–^-N**) were measured using a continuous flow-injection analyzer (Autoanalyzer 3 SEAL, Bran and Luebbe, Hamburg, Germany). Total nitrogen (TN) content was determined using an automatic Kjeldahl nitrogen analyzer (NKB3100, Shanghai Yihong Analytical, China). Organic nitrogen (ON) content was calculated by subtracting ammonium nitrogen (NH**_4_^+^-N**) from TN. Organic carbon (OC) concentration was determined using an elemental carbon analyzer (TOC-VCHS total organic carbon analyzer, Shimadzu Corporation, Kyoto, Japan). Total phosphorus (TP) was analyzed following Mo-Sb colorimetric method with an UV-Vis spectrophotometer (T-6m, RUNQEE, China). Phosphate phosphorus (PO_4_^3–^-P) was measured with a continuous flow analyzer (SAN + + , Skalar, Netherlands).

### 2.3 DNA extraction

Metagenomic DNA from each sample was extracted from 0.5 g of soil or sediment using a FastDNA^®^Spin kit for soil (MP Biomedicals, Santa Ana, CA, USA) according to manufacturer’s instructions. DNA quality and concentration were determined by 1.0% agarose gel electrophoresis and a NanoDrop^®^ ND-2000 spectrophotometer (Thermo Scientific Inc., USA).

### 2.4 16S rRNA gene sequencing

The hypervariable region V3-V4 of the bacterial 16S rRNA gene was amplified with primer pair 515F (5′- GTGYCAGCMGCCGCGGTAA-3′) and 926R (5′- CCGYCAATTYMTTTRAGTTT-3′) (Liu et al., 2016) by using an ABI GeneAmp^®^ 9700 PCR thermocycler (ABI, CA, USA). The PCR reaction mixture comprised 4 μL 5 × FastPfu buffer, 2 μL 2.5 mM dNTPs, 0.8 μL each primer (5 μM), 0.4 μL FastPfu polymerase, 10 ng template DNA, and ddH_2_O to a final volume of 20 μL. PCR amplification cycling conditions were as follows: initial denaturation at 95^°^C for 3 min, followed by 27 cycles of denaturation at 95^°^C for 30 s, annealing at 55^°^C for 30 s, and extension at 72^°^C for 45 s, with final extension at 72^°^C for 10 min, and the cycle ends at 4^°^C. All samples were amplified in triplicate. The PCR product was extracted from 2% agarose gel and purified using the AxyPrep DNA gel extraction kit (Axygen Biosciences, Union City, CA, United States) according to manufacturer’s instructions and quantified using Quantus™ fluorometer (Promega, Madison, WI, United States). Purified amplicons were pooled in equimolar amounts and paired-end sequenced on an Illumina MiSeq PE300 platform (Illumina, San Diego, CA, United States) by Majorbio Bio-Pharm Technology Co., Ltd. (Shanghai, China).

### 2.5 Metagenome sequencing

The genomic DNA obtained above was fragmented to approximately 300 bp by Covaris M220 (Gene Company Limited, China) for paired-end library construction using TruSeq™ DNA sample prep kit (Illumina, San Diego, CA, United States). Adapter ligation, cleanup, and enrichment were performed using NEXTFLEX Rapid DNA-Seq kit (Bio Scientific, United States). Paired-end sequencing was performed on Illumina Hiseq Xten platform (Illumina Inc., San Diego, CA, United States) at Majorbio Bio-Pharm Technology Co., Ltd. (Shanghai, China) using HiSeq 3000/4000 PE cluster kit and HiSeq 3000/4000 SBS kit according to the manufacturer’s instructions.^2^

### 2.6 Data processing

16S amplicon sequencing raw data was demultiplexed and quality-filtered using Trimmomatic v0.38 (Bolger et al., 2014) and assembled using FLASH v1.2.7 (Tanja and Salzberg, 2011). The maximum mismatch ratio of the overlap region was 0.2. Chimeric sequences were detected and removed using USEARCH tool with the UCHIME algorithm (Edgar et al., 2011). Operational taxonomic units (OTUs) were clustered with a 97% similarity cutoff using USEARCH v7.^3^ The taxonomy of each OTU was assigned by RDP classifier v2.2 against the Silva reference database v138 with a confidence threshold of 70% (Wang et al., 2007). Chloroplast and archaea-related sequences were manually removed. Additionally, the OTUs containing only one sequence (singleton OTUs) were removed. To avoid the bias resulting from different sequencing depths, all samples were rarefied to 20,000 sequences, which still yielded an average Good’s coverage higher than 99.5%.

Adapter sequences were stripped from the 3′ and 5′ ends of paired end Illumina reads using SeqPrep.^4^ Low-quality reads (e.g., length < 50 bp, quality value < 20, or containing N bases) were removed using Sickle.^5^ Metagenomic data were assembled using MEGAHIT v1.1.1 with default parameter setting (Li D. et al., 2015). Contigs with a length of 300 bp or more were used for further gene prediction and annotation. Open reading frames (ORFs) from each assembled contig were predicted using MetaGene.^6^ ORFs exceeding 100 bp were extracted and translated into amino acid sequences using the NCBI translation table.^7^ A non-redundant gene catalog was constructed using CD-HIT^8^ at the protein level with 95% sequence identity and 90% coverage. High-quality reads were mapped to the non-redundant gene catalogs to calculate gene abundance with 95% identity using SOAPaligner.^9^ Representative sequences of non-redundant gene catalog were aligned to the NCBI NR database using BLASTp v2.2.31 + with an e-value cutoff of 1e^–5^ for taxonomic annotation. Species identification was performed using the taxonomic information database linked to the NR database, followed by the computation of species abundance based on the sum of gene abundances. The functions of amino acid sequences were predicted using BLASTp similarity search against the Kyoto Encyclopedia of Genes and Genomes (KEGG) Orthologs (KO) database with an e-value cutoff of 1e^–5^. The KO numbers associated with ecological functions, including carbon fixation, carbon degradation, nitrification, denitrification, sulfur oxidation, and sulfate reduction, were utilized to define gene sets, followed by NR species annotation.

### 2.7 Statistical analysis

Based on the OTU information, rarefaction curves and alpha diversity indices, including observed OTUs, Chao1 richness, Shannon index, and Good’s coverage, were calculated with Mothur v1.30.1 (Schloss et al., 2009). The similarities among the microbial communities in different samples were determined by principal component analysis (PCA) based on Bray-Curtis dissimilarity using Vegan v2.5-3 package.^10^ Detrended correspondence analysis (DCA) was performed based on the abundance of species (i.e., OTUs identified based on 97% DNA sequence similarity) in the samples. Determined by the value (< 3.5) of the first length of DCA gradient, redundancy analysis (RDA) in Vegan package was further conducted to investigate the correlation between bacterial communities and environmental factors. Canonical correspondence analysis (CCA) was performed when the value of DCA1 was higher than 3.5. The statistical significance of the relationship was assessed by the Envfit analysis in Vegan package. The average values of environmental factors were expressed as mean ± standard deviation and differences were evaluated by one-way analysis of variance using SPSS v.20.0 software (SPSS Inc., Chicago, IL, United States). A *p*-value < 0.05 was considered statistically significant. The co-occurrence networks were constructed to explore the internal community relationships across the samples (Barberán et al., 2012) as well as the effects of environmental factors on bacterial community compositions (Zhu et al., 2018). A correlation between two nodes was considered to be statistically robust if the Spearman’s correlation coefficient over 0.5 or less than –0.5, and the *p*-value less than 0.05. KEGG pathway enrichment analysis was performed to identify the major metabolic pathways in different habitats by calculating FPKM values on the Majorbio Cloud platform.^11^ Statistically significant difference was calculated with the Wilcoxon rank-sum test in the STAMP software (Parks et al., 2014).

## 3 Results

### 3.1 Diversity and composition of bacterial communities revealed by 16S rRNA gene sequencing

A total of 779,052 clean reads ranging from 37,966 to 92,079 among the 12 samples were obtained for further analysis, with an average length of 396 bp. There was a total of 3,248 OTUs obtained at a cutoff level of 97%. Good’s coverage estimates for the 16S rRNA gene in each sample were higher than 99% (Table 2). Furthermore, rarefaction curve analysis showed overall saturation of diversity for the 16S rRNA gene (Supplementary Figure S1), indicating that the sequencing depth was sufficient to saturate bacterial diversity recovery in all samples. Shannon diversity index ranged from 4.15 to 5.48, with the highest and lowest values found in samples So5 and Sed2, respectively. Shannon index values were found to be higher in pristine soils (i.e., So1–So6) than in marine sediments (i.e., Sed1–Sed3). Ornithogenic soil sample Or1 not only showed low Shannon diversity index value but also exhibited the lowest Chao 1 estimator value, indicating low richness of bacterial community in ornithogenic soil than in the other three habitats.

**TABLE 2:** Bacterial diversity and richness estimates based on 97% OTU clusters.

Sample	OTUs	Shannon	Simpson	Ace	Chao 1	Coverage
IT1	1,116	4.94	0.022	1231.144	1272.45	0.998
IT2	889	4.18	0.057	1001.913	1023.33	0.998
Sed1	871	4.70	0.042	956.435	976.00	0.998
Sed2	839	4.15	0.061	951.952	990.53	0.997
Sed3	741	4.72	0.025	884.298	889.68	0.995
Or1	488	4.46	0.025	518.794	523.00	0.998
So1	1,143	5.45	0.010	1242.903	1249.99	0.996
So2	657	4.74	0.018	714.954	739.08	0.998
So3	1,121	5.01	0.020	1233.987	1275.67	0.997
So4	1,007	5.15	0.014	1088.692	1099.39	0.997
So5	1,108	5.48	0.010	1234.002	1283.78	0.996
So6	1,023	5.30	0.016	1128.916	1143.75	0.996

**FIGURE 2:**
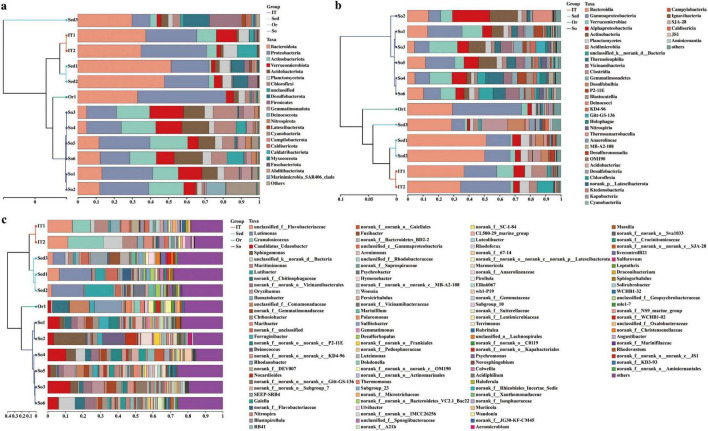
Bacterial community compositions at phylum **(A)**, class **(B),** and genus **(C)** levels across all samples. Others indicate sum of taxonomic groups representing less than 0.5% of total bacterial sequences in the four habitats. So, pristine soil; Or, ornithogenic soil; IT, intertidal sediment; Sed, marine sediment.

Besides taxonomically unaffiliated bacteria (including no rank and unclassified bacteria), 46 bacterial phyla were detected in the 12 samples. Bacterial taxa exhibiting a relative abundance greater than 1% of the total sequences at various taxonomic levels were designated as dominant. *Bacteroidota* (25.8% in average; mainly by *Bacteroidia*), *Proteobacteria* (25.4%; mainly by *Gamma*- and *Alphaproteobacteria*), *Verrucomicrobiota* (7.9%; mainly by *Verrucomicrobiae*), and *Planctomycetota* (4.6%; mainly by *Planctomycetes*) were dominant across all samples (Figure 2). Additionally, *Acidobacteriota*, *Actinobacteriota*, *Chloroflexi*, *Firmicutes*, and *Gemmatimonadota* were frequently observed in all samples. However, much higher relative abundance of *Bacteroidota* was observed in marine sediments (Sed1–Sed3; 42.9%), intertidal sediments (IT1 and IT2; 36.1%), and ornithogenic soil (Or1; 32.9%) than in pristine soils (So1–So6; 9.8%). In addition, *Desulfobacterota* was exclusively dominant in marine sediments (9.9%). Sequences affiliated with *Campilobacterota*, *Caldatribacteriota*, *Fusobacteriota* as well as Marinimicrobia_SAR406_clade were exclusively detected in low abundance in marine-influenced samples, such as marine and intertidal sediments. Contrarily, more abundant *Actinobacteriota* (represented by *Actinobacteria* and *Thermoleophilia*) was detected in pristine soils (17.6%) than in marine sediments (2.6%), intertidal sediments (8.0%), and ornithogenic soil (0.4%). Similarly, *Gemmatimonadota* (represented by *Gemmatimonadetes*) was dominant in pristine soils (3.9%) but rare in the other three habitats (< 0.5%). Meanwhile, higher abundance of *Verrucomicrobiae* within the *Verrucomicrobiota* and *Blastocatellia* within the *Acidobacteriota* was found in pristine soils (11.3 and 2.3%, respectively) than in marine sediments (2.1 and < 0.01%, respectively). In addition, *Vicinamibacteria* within the *Acidobacteriota* was more abundant in terrestrial samples (i.e., pristine and ornithogenic soils; 4.4%) than in marine samples (i.e., intertidal and marine sediments; 0.04%). *Abditibacteriota* was exclusively detected in low abundance in terrestrial samples, and *Deinococcota* was absent in marine sediments. Though a large number of cyanobacterium-like sequences (2.5%) were detected in samples especially in pristine soils, most of them actually belonged to chloroplasts. Cyanobacteria, including *Sericytochromatia* and *Vampirivibrionia*, were exclusively detected in terrestrial samples in small amounts (< 0.1%). *Gammaproteobacteria* was more abundant in ornithogenic soil (44.6%) followed by intertidal sediments (26.9%), and marine sediments (14.4%) than in pristine soils (12.9%). Irrespective of phylum, class, or genus levels, bacterial communities in pristine soils usually formed a cluster separate from those in marine-influenced samples (Figure 2).

At the genus level, sequences within *Clostridium_ sensu_stricto_13*, *Flavobacterium*, *Ilumatobacter*, *Luteolibacter*, *Nocardioides*, *Parafrigoribacterium*, *Rhodoferax*, an unaffiliated *Pirellulaceae* group, an unaffiliated *Rubinisphaeraceae* group, an unaffiliated *Saprospiraceae* group, and an unaffiliated KD4-96 group were detected in all samples. However, there was no dominant bacterial group across all samples. *Ilumatobacter* and *Maritimimonas* were abundant (2.0–3.1%) in marine samples, whereas *Ferruginibacter*, *Candidatus* Udaeobacter, an unaffiliated *Chitinophagaceae* group, and an unaffiliated *Comamonadaceae* group were dominant (2.8–6.9%) in terrestrial samples. On the land, *Chthoniobacter*, *Nocardioides*, *Sphingomonas*, an unaffiliated Subgroup_7 group, an unaffiliated *Vicinamibacterales* group, and an unaffiliated KD4-96 group were dominant (2.0–6.5%) in pristine soils, whereas *Rhodanobacter*, *Rhodoferax*, an unaffiliated *Xanthomonadaceae* group, an unaffiliated C0119 group, and an unaffiliated *Oxalobacteraceae* group were abundant (1.7–16.8%) in ornithogenic soil. In marine habitats, *Blastopirellula*, *Granulosicoccus*, *Maribacter*, and an unaffiliated *Rhodobacteraceae* group were dominant (1.6–18.2%) in intertidal sediments, whereas *Fusibacter*, *Lutibacter*, *Luteimonas*, *Marinifilum*, SEEP-SRB4, Subgroup_23, and an unaffiliated *Bacteroidetes*_BD2-2 group were dominant (1.9–16.5%) in marine sediments.

Sequences affiliated with the *Desulfobulbus*, *Desulfoconvexum*, *Dethiosulfatibacter*, JTB215, *Labilibacter*, MSBL3, *Pelolinea*, SEEP-SRB1, Sva0081_sediment_group, UCG-012, livecontrolB21, an unaffiliated *Calditrichaceae* group, an unaffiliated *Lachnospiraceae* group, an unaffiliated *Marinilabiliaceae* group, an unaffiliated *Mycoplasmataceae* group, an unaffiliated SB-5 group, an unaffiliated SG8-4 group, an unaffiliated *Sedimenticolaceae* group, an unaffiliated WCHB1-41 group, an unaffiliated BD2-11_terrestrial_group, an unaffiliated JS1 group, an unaffiliated PAUC43f_marine_benthic_group, an unaffiliated WCHB1-81 group, an unaffiliated *Marinimicrobia*_SAR406_clade group, and an unaffiliated *Christensenellaceae* group were detected in marine sediments only. Simultaneously, sequences related to *Abditibacterium*, *Acidiphilium*, *Caulobacter*, *Chthonomonas*, DEV114, *Edaphobaculum*, *Flavisolibacter*, *Flavitalea*, *Hyphomicrobium*, MND1, *Novosphingobium*, *Phenylobacterium*, *Pseudolabrys*, *Schlesneria*, *Zavarzinella*, mle1-7, an unaffiliated A0839 group, an unaffiliated *Acetobacteraceae* group, an unaffiliated BIrii41 group, an unaffiliated *Caldilineaceae* group, an unaffiliated *Devosiaceae* group, an unaffiliated JG30-KF-CM45 group, an unaffiliated *Roseiflexaceae* group, an unaffiliated WD2101_soil_group, an unaffiliated *Chitinophagales* group, an unaffiliated Lineage_IV group, an unaffiliated RBG-13-54-9 group, an unaffiliated *Acidimicrobiia* group, an unaffiliated OLB14 group, an unaffiliated P2-11E group, an unaffiliated *Acetobacteraceae* group, an unaffiliated *Blastocatellaceae* group, an unaffiliated *Geodermatophilaceae* group, and an unaffiliated *Pedosphaeraceae* group were exclusively observed in pristine soils.

Co-occurrence networks were constructed based on Spearman’s correlations among top 50 genera (Supplementary Figure S2). A general positive correlation was observed among the bacterial genera, with more edges representing positive correlations than negative ones. A total of 48 nodes linked by 131 edges comprised the terrestrial bacterial community network, and 48 nodes linked by 217 edges comprised the marine bacterial community network. These results showed that the marine bacterial community was more complex and stable than the terrestrial bacterial community.

**FIGURE 3:**
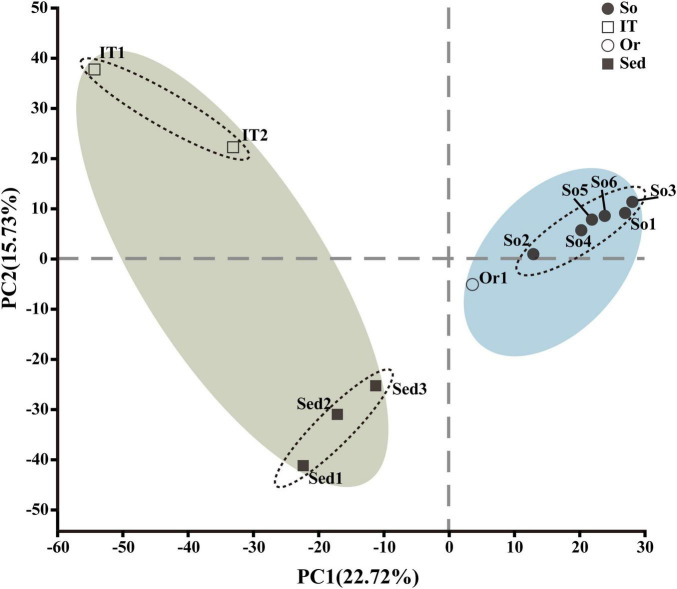
Principal component analysis (PCA) depicting the bacterial diversity patterns across 12 samples based on OTUs assigned at 97% sequence similarity. So, pristine soil; Or, ornithogenic soil; IT, intertidal sediment; Sed, marine sediment.

PCA was conducted at the OTU level to illustrate bacterial diversity patterns across all samples (Figure 3). Consistent with the above findings (Figure 2C), clear variation between the marine (i.e., Sed1–Sed3 and IT1–IT2) and terrestrial (i.e., Or1 and So1–So6) samples was found along the first axis (*p* = 0.003), indicating distinct microbiota compositions in terrestrial and marine ecosystems. Furthermore, the marine sediment samples (Sed1–Sed3) were separated from the intertidal sediment samples (IT1–IT2) by the second axis, suggesting a difference in microbiota composition between intertidal and marine sediment habitats.

### 3.2 Effect of environmental factors on bacterial community composition

The environmental factors of the 12 samples are shown in Table 3. Much higher contents of ammonium nitrogen (NH_4_^+^**-N**), ON, TN, phosphate (PO_4_^3^**^–^**), and TP were detected in ornithogenic soil than in the other three habitats. The lowest pH value was found in ornithogenic soil. The pH values in marine samples tended to be alkaline (pH 8.35 ± 0.56), whereas those in terrestrial samples were weakly acidic (pH 6.30 ± 0.42). The marine samples usually contained higher OC content (7.29 ± 0.15%) than terrestrial samples (1.55 ± 1.27%).

**TABLE 3:** Environmental characteristics of study samples.

Sample	NO_3_^–^-N (μg/g)	NH_4_^+^-N (μg/g)	ON (mg/g)	TN (mg/g)	OC (%)	PO_4_^3–^-P (μg/g)	TP (mg/g)	pH
Or1	9.16	101.20	14.21	14.31	3.02	72.87	22.35	5.77
So1	7.66	12.94	1.19	1.20	1.13	2.64	0.60	6.17
So2	7.94	15.50	1.13	1.15	1.50	2.86	0.20	6.25
So3	7.99	10.56	0.52	0.53	1.14	5.92	1.68	6.54
So4	7.02	10.93	0.97	0.98	0.77	1.07	0.36	6.64
So5	6.94	12.04	0.86	0.87	2.08	0.92	0.46	6.35
So6	7.76	15.60	1.50	1.51	1.22	1.10	0.41	6.37
IT1	7.54	11.25	1.65	1.66	7.93	1.56	0.56	8.35
IT2	6.58	10.75	1.22	1.23	5.36	1.88	0.69	8.87
Sed1	7.83	13.26	0.67	0.69	7.59	3.11	1.17	8.41
Sed2	7.45	12.79	0.59	0.60	7.85	4.93	0.85	8.58
Sed3	9.26	13.13	1.74	1.76	7.72	3.74	1.10	7.55

ON, organic nitrogen; TN, total nitrogen; OC, organic carbon; TP, total phosphorus.

**FIGURE 4:**
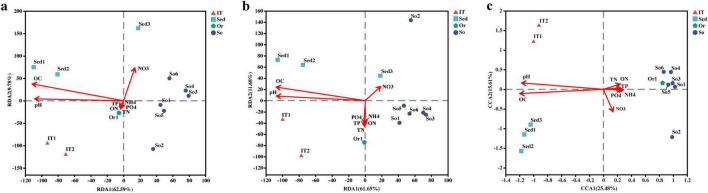
Correlation between environment factors and bacterial community compositions at phylum **(A)**, class **(B),** and genus **(C)** levels. So, pristine soil; Or, ornithogenic soil; IT, intertidal sediment; Sed, marine sediment.

The relationships between environmental factors and bacterial community structure at the phylum and class levels were shown by RDA (Figures 4a,b). The first two RDA axes could explain more than 72% of the total variance in bacterial community composition. Envfit analysis showed that OC (*r*^2^ = 0.8639, *p* = 0.002) and pH (*r*^2^ = 0.7182, *p* = 0.002) were the main factors affecting the bacterial community composition in the 12 samples at the phylum level. Furthermore, OC exhibited positive correlation with pH value (*r*^2^ = 0.7182, *p* = 0.0005). At the genus level, the relationship was exhibited by CCA (Figure 4C) because of the first DCA axis being higher than 4. In contrast, the first two CCA axes only explained 41.1% of the total variance in the bacterial community composition, although OC and pH were still the main factors influencing bacterial communities.

**FIGURE 5:**
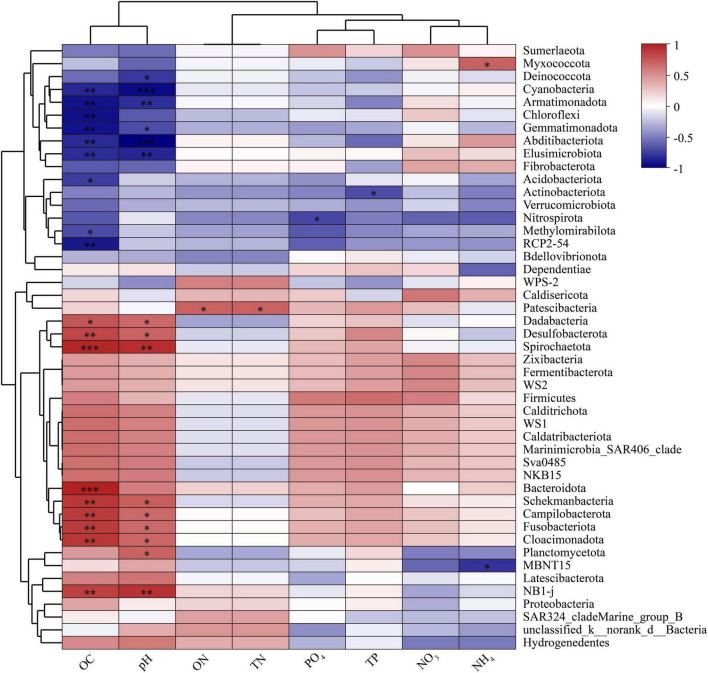
Spearman’s correlation between relative abundance of bacteria at phylum level and environmental factors (*p < 0.05; **p < 0.01; ***p < 0.001).

The Spearman’s correlations between the relative abundance of bacteria at the phylum level and the environmental factors were calculated (Figure 5). The relative abundance of *Campilobacterota*, *Cloacimonadota, Dadabacteria, Desulfobacterota, Fusobacteriota*, NB1-j, *Schekmanbacteria*, and *Spirochaetota* was positively correlated with OC and pH, whereas *Abditibacteriota, Armatimonadota, Elusimicrobiota*, and *Gemmatimonadota* abundance was negatively correlated with OC and pH. Among the bacterial phyla dominant across all samples, *Bacteroidota* and *Planctomycetota* were positively correlated with OC and pH, respectively. *Acidobacteriota* and *Chloroflexi* were negatively correlated with OC. In addition, *Actinobacteriota* and *Deinococcota* were negatively correlated with TP and pH, respectively.

**FIGURE 6:**
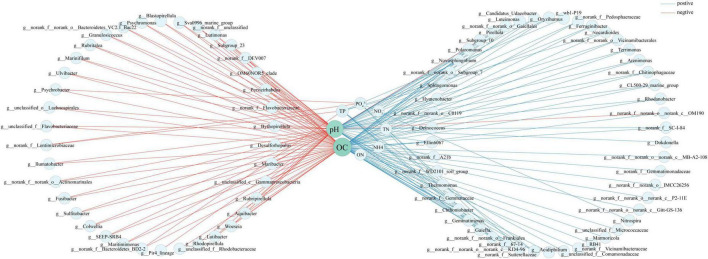
Network analysis showing interactions between environmental factors and bacteria at genus level. Red and blue lines indicate positive and negative correlations, respectively. Environmental factor node size is proportional to extent of correlation.

A Spearman correlation network was constructed for taxa with the absolute value of correlation coefficient greater than 0.5 at the genus level (Figure 6). OC and pH were most closely associated with different bacterial taxa, correlating with 79 and 67 bacterial genera, respectively. A total of 43 and 34 bacterial genera were negatively and positively correlated with OC, respectively. Meanwhile, there were 31 and 35 bacterial genera negatively and positively correlating with pH, respectively.

### 3.3 Metagenomic profiling

Total 120.49 gigabases of clean reads were obtained from 12 samples with an average 97.33% (clean reads percent in raw reads) high quality. By predicting the ORFs of the assembled contigs, a total of 20,005,448 genes and 15,202,969 non-redundant genes were found, with an average length of 440 bp and 495 bp, respectively. Genes were assigned the taxonomic classification of their top hits against the NCBI NR database (including *Bacteria*, *Archaea*, *Eukaryota*, and viruses). Taxonomic annotation of metagenomic sequencing data showed that *Bacteria* (98.60–99.37% of total reads in samples) prevailed relatively to *Eukaryota*, *Archaea*, and viruses in all metagenomes (Table 4). *Proteobacteria*, *Actinobacteria*, *Bacteroidetes*, *Chloroflexi*, *Acidobacteria*, *Firmicutes*, *Gemmatimonadetes*, *Verrucomicrobia*, *Planctomycetes*, and *Nitrospirae* were abundant (> 1%) in metagenomic data. The marine and terrestrial samples carried different microbial assemblages. For example, more abundant *Actinobacteria* (42.6% in average), *Chloroflexi* (8.5%), *Acidobacteria* (4.5%), and *Gemmatimonadetes* (3.6%) were observed in terrestrial samples than in marine samples (accounting for 13.3, 1.5, 0.8, and 0.6%, respectively). On the contrary, more abundant *Bacteroidetes* (23.9%) and *Proteobacteria* (45.3%) were detected in marine samples than in terrestrial samples (5.0 and 22.5%, respectively; Supplementary Table S1).

**TABLE 4:** Overall phylogenetic structure of microbial communities in 12 investigated samples.

Station	Bacteria (%)	Eukaryota (%)	Archaea (%)	Viruses (%)	Unclassified (%)
IT1	98.90	0.41	0.59	0.01	0.09
IT2	99.17	0.25	0.50	0.01	0.07
Sed1	98.06	0.34	0.41	1.12	0.08
Sed2	99.02	0.21	0.66	0.05	0.05
Sed3	98.48	0.33	1.04	0.02	0.12
Or1	99.37	0.23	0.32	0.04	0.04
So1	99.18	0.17	0.51	0.02	0.13
So2	99.32	0.44	0.18	0.03	0.03
So3	99.09	0.13	0.65	0.01	0.13
So4	98.42	0.42	0.96	0.02	0.17
So5	98.91	0.27	0.65	0.01	0.15
So6	98.60	0.14	1.10	0.01	0.14

**FIGURE 7:**
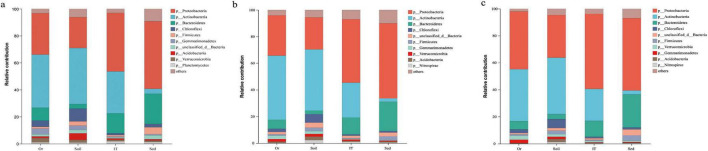
Composition of bacteria contributing to carbon **(A)**, nitrogen **(B),** and sulfur **(C)** metabolism in different habitats at phylum level. Others indicate sum of phyla representing less than 0.5% of total bacterial sequences in the four habitats. So, pristine soil; Or, ornithogenic soil; IT, intertidal sediment; Sed, marine sediment.

**FIGURE 8:**
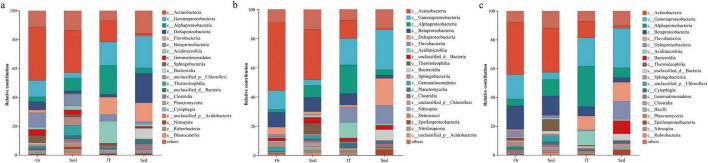
Composition of bacteria contributing to carbon **(A)**, nitrogen **(B),** and sulfur **(C)** metabolism in different habitats at class level. Top 20 classes are shown. Others indicate sum of classes representing less than 0.5% of total bacterial sequences in the four habitats. So, pristine soil; Or, ornithogenic soil; IT, intertidal sediment; Sed, marine sediment.

*Proteobacteria* (36.9% in average; including *Alpha-, Beta*-,*Gamma-*, and *Deltaproteobacteria*), *Actinobacteria* (28.9%; including *Actinobacteria*, *Acidimicrobiia*, and *Thermoleophilia*), and *Bacteroidetes* (12.5%; including *Flavobacteriia*) dominated in bacteria involved in carbon metabolism across all samples (Figures 7A, 8A). *Proteobacteria* (22.7–50.8%; represented by *Gammaproteobacteria*) and *Bacteroidetes* (3.6–22.3%; represented by *Flavobacteriia*) showed an increasing trend from pristine soils, ornithogenic soil, intertidal sediments to marine sediments, whereas *Actinobacteria* (3.6–41.8%; represented by *Thermoleophilia*) and *Acidobacteria* (0.8–4.8%) exhibited a reverse trend. *Chloroflexi* was much more abundant in pristine soils (9.3%) than in the other three habitats (1.3–4.6%). The classes *Actinobacteria*, *Betaproteobacteria*, and *Gemmatimonadetes* were much more abundant in terrestrial environments (33.4, 9.0, and 3.6%, respectively) than in marine environments (8.3, 3.2, and 0.7%, respectively). In addition, *Sphingobacteriia* (5.3%) was more abundant in ornithogenic soil than in the other three habitats, *Alphaproteobacteria* (20.3%) and *Acidimicrobiia* (15.3%) were much more abundant in intertidal sediments than in the other three habitats, and *Deltaproteobacteria* (20.1%) and *Bacteroidia* (7.1%) were much more abundant in marine sediments than in the other three habitats. Similar results were observed in bacteria involved in nitrogen (Figures 7B, 8B) and sulfur metabolism (Figures 7C, 8C). However, unlike carbon metabolism, *Epsilonproteobacteria* was exclusively dominant in bacteria involved in nitrogen and sulfur metabolism (3.4 and 2.9%, respectively) in marine sediments.

### 3.4 Ecological functional properties in different habitats

Based on KEGG annotation of environmental metagenome, most metabolic pathways found in marine samples could be detected in terrestrial samples (Supplementary Figure S3). Meanwhile, differences in metabolic pathways were observed between marine and terrestrial ecosystems. For example, some of genes related to benzoate, xylene, and dioxin degradation and ansamycin biosynthesis were exclusively observed in terrestrial samples, whereas some of genes involved in glycosaminoglycan and aminobenzoate degradation, phenylalanine, tyrosine, and tryptophan biosynthesis, biotin metabolism, and xenobiotic biodegradation and metabolism were found in marine samples only. Overall, terrestrial samples exhibited more diverse metabolic pathways than marine samples.

**FIGURE 9:**
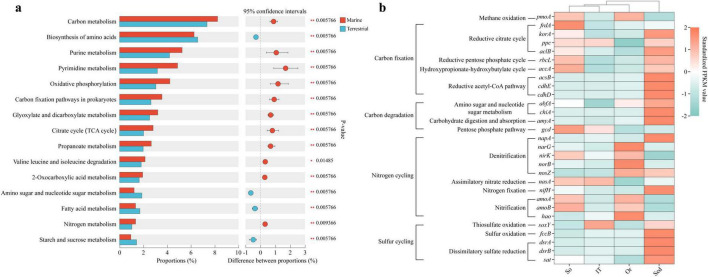
Differences in functional properties based on metagenome KEGG annotation. **(A)** KEGG categories differing significantly between terrestrial and marine ecosystems. **(B)** Heatmap showing differences in relative abundance of key function genes associated with carbon, nitrogen, and sulfur metabolism in four habitats. So, pristine soil; Or, ornithogenic soil; IT, intertidal sediment; Sed, marine sediment.

A total of 162 microbial metabolic pathways were detected in environmental metagenomes (Supplementary Table S2). Significant differences in relative abundance of functional genes related to 92 metabolic pathways were found between marine and soil samples: genes involved in 72 metabolic pathways displayed higher abundance in terrestrial samples than in marine samples, whereas genes related to other 20 metabolic pathways were more abundant in marine samples than in terrestrial samples. Among the top 15 abundant metabolic pathways with significant difference (*p* < 0.05; Figure 9A), except for amino acid biosynthesis and amino sugar, nucleotide sugar, fatty acid, starch, and sucrose metabolism, genes related to 11 metabolic pathways, such as carbon metabolism, carbon fixation pathways in prokaryotes, and nitrogen metabolism, were much more abundant in marine samples than in terrestrial samples.

Particulate methane monooxygenase gene *pmoA* related to methane metabolism and ammonia monooxygenase gene *amoAB* related to nitrification were more abundant in terrestrial samples than in marine samples (Figure 9B). In addition, fumarate reductase gene *frdA* involved in reductive citrate cycle was much more abundant in pristine soils than in the other three habitats, whereas genes related to denitrification (e.g., *narG* and *norB*) were relatively more abundant in ornithogenic soil than in the other three habitats. However, phosphoenolpyruvate carboxylase gene *ppc* related to tricarboxylic acid (TCA) cycle was in the lowest abundance in ornithogenic soil than in the other three habitats. Genes (e.g., *lexA*, *recF*, and *uvrB*) involved in repair of DNA damage produced by ionizing radiation and ultraviolet radiation were found to be more abundant in pristine soils (Supplementary Figure S4). Meanwhile, genes (e.g., *betB*, *gltB*, *nhaH*, *osmC*, and *treS*) related to salt and osmotic stress tolerance were more abundant in terrestrial samples especially in pristine soils. Benzylsuccinate synthase gene *bssA* and benzoyl-CoA reductase gene *bcrC* were relatively more abundant in ornithogenic soil, while xylene monooxygenase gene *xylA* was in higher abundance in pristine soils. However, *xylM* was absent from the metagenomics data. Xylene monooxygenase catalyzes the oxidation of toluene and xylenes and consists of two different subunits encoded by *xylA* and *xylM* genes (Suzuki et al., 1991).

In contrast, genes related to sulfur metabolism, including sulfur oxidation (e.g., sulfur carrier protein gene *soxY* and flavocytochrome c sulfide dehydrogenase gene *fccB*) and sulfate reduction (e.g., dissimilatory sulfite reductase gene *dsrAB* and sulfate adenylyltransferase gene *sat*), were much more abundant in marine sediments and/or intertidal sediments than in the other habitats. In addition to genes related to denitrification (e.g., nitrate reductase gene *napA*) and nitrogen fixation (e.g., nitrogenase gene *nifH*), genes related to reductive acetyl-CoA pathway (e.g., acetyl-CoA synthase gene *acsB* and CO dehydrogenase-related gene *cdhDE*) and carbohydrate degradation (e.g., alpha-amylase gene *amyA* and chitinase gene *chiA*) were in high abundance in marine sediments than in the other habitats. It was noticed that genes (e.g., *cspA*, *deaD*, *hepA*, and *recQ*) related to cold adaptation were generally more abundant in marine sediments. Furthermore, single-stranded DNA-binding protein gene *ssb* was relatively more abundant in marine sediments, while TrkA domain protein gene *trkA* associated with potassium ion uptake was in higher abundance in intertidal sediments.

## 4 Discussion

### 4.1 Bacterial community and diversity

*Bacteroidota*, *Proteobacteria*, *Verrucomicrobiota*, *Plancto mycetota*, *Acidobacteriota*, *Actinobacteriota*, and *Chloroflexi*, were frequently observed across all samples. These bacterial groups have diverse metabolic pathways required for carbon and nitrogen transformation and stress response in Antarctic environments (Ganzert et al., 2011; Cameron et al., 2012; Alcántara-Hernández et al., 2014; Wei et al., 2016; Adriaenssens et al., 2017; Waschulin et al., 2022). Meanwhile, the bacterial community compositions in terrestrial samples were distinct from those in marine samples (Figures 2, 3). Compared to five phyla (i.e., *Abditibacteriota, Elusimicrobiota, Fibrobacterota, Methylomirabilota*, and *Sumerlaeota*) exclusively detected in the terrestrial samples, a total of 16 phyla (*Caldatribacteriota, Calditrichota, Campilobacterota, Cloacimonadota, Dadabacteria, Fermentibacterota, Fusobacteriota, Hydrogenedentes*, *Marinimicrobia*_SAR406_clade, NKB15, *Schekmanbacteria, Spirochaetota*, Sva0485, WS1, WS2, and *Zixibacteria*) were observed in marine samples only (Supplementary Figure S5), suggesting more diverse bacterial taxa inhabiting marine environments than terrestrial environments. It is in line with previous studies conducted in the same region, showing that more bacterial phyla are detected in intertidal sediments than in terrestrial environments (Wang et al., 2015; Wang et al., 2016; Wang et al., 2022; Nopnakorn et al., 2023). Marine sediments can harbor the highest microbial diversity than various other Antarctic habitats (Bendia et al., 2023). This phenomenon may be due to the relatively more consistent environmental conditions (e.g., temperature, water content, nutrient, and UV irradiation) in marine ecosystem than in terrestrial ecosystem, which is helpful for survival and growth of microbes in Antarctica. More stable low temperature conditions are reported in Antarctic marine waters than in soils, potentially resulting in that the bacterial communities in the ocean are more adapted to low temperatures than those in nearby soil (van-Gestel et al., 2020). Conversely, at the OTU level (Table 2 and Supplementary Figure S6), higher bacterial diversity was observed in terrestrial samples than in marine samples. It may be attributed to the presence of variable copy numbers of 16S rRNA gene in bacterial genomes and sequence variation within closely related taxa or a genome, suggesting that OTUs provide an imperfect representation of bacterial taxa of a certain phylogenetic rank (Větrovský and Baldrian, 2013). Simultaneously, KEGG annotations of environmental metagenome revealed that terrestrial samples harbored more diverse metabolic pathways than marine samples (Supplementary Figure S3). It can be attributed to harsh and frequently disturbed environments (e.g., low temperature, low nutrient availability, high UV radiation, and frequent freeze-thaw activity) on the Antarctic land, which is helpful in the evolution of terrestrial microorganisms to adapt to various environmental stresses.

The abundance of *Actinobacteriota* decreased from pristine soils, to intertidal sediments, to marine sediments, and to ornithogenic soil. Compared to marine sediments (Franco et al., 2017), *Actinobacteria* often make up a major fraction of bacterial communities in terrestrial soils (Wang et al., 2015; Benaud et al., 2021) and intertidal sediments of the AP (Wang et al., 2016) due to their resilience and adaptability to survive under harsh circumstances (Shivlata and Satyanarayana, 2015; Nopnakorn et al., 2023). Consistent with that actinobacteria are dominant in Antarctic arid soil and their abundance decline with organic matter addition (Buelow et al., 2016), a sharp decline in relative abundance of actinobacteria was observed in ornithogenic soil compared to pristine soils in this study, indicating the influence of organic matter supplementation through penguin feces on actinobacteria in terrestrial soils. Though *Actinobacteria* dominated in intertidal sediments, differences in *Actinobacteria* diversity was observed between pristine soils and intertidal sediments. For example, compared to the dominance of genera *Ilumatobacter* and Sva0996_marine_group in intertidal sediments, the genera *Conexibacter*, *Gaiella*, *Oryzihumus*, *Marmoricola*, *Nocardioides*, and *Solirubrobacter* were dominant in pristine soils. Additionally, the genera *Ilumatobacter* and Sva0996_marine_group were dominant in marine sediments, suggesting the survival and thriving ability of such specific actinobacterial genera in marine environments.

*Gemmatimonadota* was much more abundant in pristine soils. As budding bacteria, *Gemmatimonadetes* are well-adapted to low-moisture environments and possess strong tolerance to various harsh environments (DeBruyn et al., 2011; Nopnakorn et al., 2023). With the ability to reduce nitrite to nitric oxide in soil (Helen et al., 2016), *Gemmatimonadaceae* (e.g., genera *Gemmatimonas* and *Roseisolibacter*) predominated in *Gemmatimonadota*-related sequences. Members of the *Gemmatimonas* not only have the capability to utilize dissolved organic substrates and harvest light energy to grow (Zeng et al., 2015; Zeng et al., 2021), but are also capable of reducing the potent greenhouse gas N_2_O under anaerobic and aerobic conditions (Chee-Sanford et al., 2019). *Gemmatimonas* has been detected in limnetic microbial mats and soils on King George Island (Sampaio et al., 2017; Valdespino-Castillo et al., 2018). The genus *Roseisolibacter* is more abundant in healthy rhizosphere soil than in diseased rhizosphere soil (Wu et al., 2024). However, the ecological roles of *Roseisolibacter* in environments are poorly understood, as only one species *Roseisolibacter agri* AW1220^T^ has been isolated from agricultural soil as a pure culture available for detailed studies (Pascual et al., 2018). The genera *Gemmatimonas* and *Roseisolibacter* have been proposed to be biomarkers of the entire soil profile in alpine meadow in response to climate warming (Zhou et al., 2023), suggesting that they can also be biomarkers of the whole soil profile of polar regions.

In contrast, orders *Desulfatiglandales* (e.g., *Desulfatiglans*), *Desulfobacterales* (e.g., *Desulfobacter*, *Desulfobacterium*, *Desulfoconvexum*, *Desulfofrigus*, SEEP-SRB1, and Sva0081_ sediment_group), and *Desulfobulbales* (e.g., *Candidatus*_ Electrothrix and *Desulfobulbus*) within the *Desulfobacterota* were exclusively dominant in marine sediments (9.9%) but rare in the other three habitats (≤ 0.3%). These sulfate-reducing bacterial genera have been proposed to form syntrophic consortia with anaerobic methanotrophic archaea in marine sediments (Ozuolmez et al., 2015; Calvo-Martin et al., 2022; Yu et al., 2022). Simultaneously, the order *Geobacterales* (e.g., genus *Geobacter*) within the *Desulfobacterota* was usually present in terrestrial samples and absent in marine sediments. *Geobacter* spp. are dissimilatory metal and sulfur reducing bacteria that can gain energy by reducing sulfur or sulfate to hydrogen sulfide with OC oxidation (Lovley et al., 1993; Caccavo et al., 1994). This genus is commonly found in anaerobic environments and is one of the most dominant nitrogen fixers present in glacier forefield soils (Nash et al., 2018). The detection of *Geobacter*-related sequences in terrestrial samples implies their potential in sulfur reduction and nitrogen fixation in Antarctic soils. As an electroactive microorganism widely occurred in soil, *Geobacter* plays a key role in regulating emissions and biogeochemical cycling of soil-derived greenhouse gasses, such as carbon dioxide (CO_2_), methane (CH_4_), and nitrous oxide (N_2_O), through redox reactions under anaerobic conditions (Li and Zhou, 2020). Key diazotrophs in Arctic forefields, including *Geobacter*, *Frankia*, *Polaromonas*, and *Bradyrhizobium*, have been found to be metabolically diverse (Nash et al., 2018). In this study, these genera were usually present in the terrestrial soils and absent in the marine sediments.

Though belonging to the terrestrial ecosystem in the Fildes region, ornithogenic soil always formed a separate line from the cluster of pristine soils at different taxonomic levels (Figure 2), indicating a difference in bacterial community composition between ornithogenic and pristine soils. Furthermore, ornithogenic soil showed lower values of Shannon diversity index and Chao 1 estimator than pristine soils (Table 2), indicating that ornithogenic soil harbored less diverse bacterial composition than pristine soils. It is supported by the finding that all bacterial phyla detected in ornithogenic soil (22 phyla) could be observed in pristine soils (30 phyla; Supplementary Figure S3). Compared to pristine soils dominated (≥ 5%) by *Proteobacteria* (mainly by *Gamma-* and *Alphaproteobacteria*), *Actinobacteriota*, *Verrucomicrobiota*, *Acidobacteriota*, *Bacteroidota*, and *Chloroflexi*, ornithogenic soil was absolutely dominated by *Proteobacteria* (mainly by *Gammaproteobacteria*) and *Bacteroidota*. At the genus level, *Dokdonella*, *Leptothrix*, *Polaromonas*, *Rhodanobacter*, *Rhodoferax*, *Thermomonas*, a no rank *Chitinophagaceae* group, and an unclassified *Comamonadaceae* group together accounted for 48.1% of the bacterial community composition in ornithogenic soil. Both *Dokdonella* and *Thermomonas* are aerobic gammaproteobacterium and are frequently isolated from soil (Wang L. et al., 2014; Villamil et al., 2021). Nitrogen fertilizers can increase abundances of the bacterial denitrifiers *Dokdonella* and *Thermomonas* (Villamil et al., 2021). Within the *Gammaproteobacteria*, the genus *Rhodanobacter* is one of the most abundant genera in soils impacted by marine birds (Ramírez-Fernández et al., 2021) and connected with denitrification of soil (Kostka et al., 2012). This genus was the most abundant genus (15.6%) in ornithogenic soil sample used in this study. The genus *Leptothrix* within the family *Comamonadaceae* of *Betaproteobacteria* has been isolated from the intestine of insects (Wang W. W. et al., 2014) and has been found to be dominant in wastewater generated from hospitals (Tang et al., 2021). Biogenic iron oxides formed by *Leptothrix* spp. play an important role in environmental and engineered systems for phosphate removal (Buliauskaitë et al., 2020). Within the same family, *Polaromonas* and *Rhodoferax* spp. are generalist and ubiquitous in cold environments (Franzetti et al., 2013; Gladkov et al., 2024) and play roles in denitrification and nitrogen fixation in terrestrial environments (Baker et al., 2017; Nash et al., 2018; Yang et al., 2019; Foysal et al., 2022). Though the presence of an unclassified *Comamonadaceae* group (11.3%) and a no rank *Chitinophagaceae* group (9.3%) exclusively dominant in ornithogenic soil of this study is unclear, the supplementation of penguin feces could be an important factor determining their presence. Ornithogenic soil in this study was collected from Ardley Island—an important breeding area for Adélie (*Pygoscelis adeliae*) and Gentoo (*Pygoscelis papua*) penguins (Zeng et al., 2022). Antarctic krill (i.e., *Euphausia* sp.) are the main prey of pygoscelid penguins (Zeng et al., 2022). Members of *Chitinophagaceae* within the *Bacteroidota* are of special interest owing to their ability to degrade chitin (Glavina-Del-Rio et al., 2010)—the main component of the exoskeleton of crustaceans (e.g., krill). Collectively, the dominance of the above described genera indicates the influence of penguin feces on composition and function of the bacterial community in Antarctic soils due to the supplementation of high contents of nitrogenous (e.g., ammonium nitrogen NH**_4_^+^-N**) and phosphorous (e.g., phosphate) fertilizers (Table 3) as well as food debris originated from krill (Almela et al., 2022). Additionally, it is consistent with a conception that a higher nutrient input possibly leads to a change in soil bacterial community shifting from an oligotrophic *Acidobacteria*-dominated or more diverse bacterial community to a less diverse bacterial community dominated by copiotrophic *Bacteroidetes* (Ganzert et al., 2011). Fierer et al. (2007) found that carbon supplementation led to a positive correlation between the *Bacteroidetes* abundance and carbon mineralization rates, whereas the *Acidobacteria* abundance was negatively correlated with carbon mineralization rates.

In the Fildes region, intertidal sediments generally formed a cluster separated from that of marine sediments (Figure 2), indicating a difference in bacterial community composition between intertidal sediments and marine sediments. Meanwhile, there were one and nine bacterial phyla exclusively detected in intertidal sediments and marine sediments, respectively (Supplementary Figure S5). Compared to marine sediments harboring 36 phyla, intertidal sediments contained 31 phyla, exhibiting less diverse bacterial composition. However, compared to marine sediments sharing 58.3% of the detected phyla with terrestrial samples, intertidal sediments shared 77.4% and 83.9% of bacterial phyla with terrestrial samples and marine sediments, respectively. The results not only suggest a transitional boundary of intertidal sediments between terrestrial and marine environments, but also reveal more similar bacterial community composition in intertidal sediments and marine sediments than in terrestrial soils, supporting that intertidal sediments belong to marine ecosystem.

### 4.2 Environmental variables affecting bacterial community composition

In this study, OC and pH were found to have a significant impact on the bacterial community composition in various habitats of the Fildes region (Figures 4–6). For example, more abundant *Acidobacteriota* was detected in pristine soils (10.1%) than in the other three habitats (1.4–2.7%). *Acidobacteriota* was negatively correlated with OC, consistent with the finding that *Acidobacteria* are abundant in Antarctic soils with low carbon and nitrogen contents (Ganzert et al., 2011). The abundance of *Acidobacteriota* is negatively correlated with carbon mineralization rates (Fierer et al., 2007). In contrast, *Bacteroidota* was positively correlated with OC. From pristine soils, ornithogenic soil, intertidal sediments to marine sediments, the OC content increased from 1.3 to 7.7%, and the relative abundance of *Bacteroidetes* increased from 9.8 to 42.9% (Table 3; Figure 2A). Additional carbon can lead to a positive correlation between the *Bacteroidetes* abundances and carbon mineralization rates (Fierer et al., 2007), implying an important role of *Bacteroidota* in the Fildes region to degrade polymeric organic matter and provide low molecular weight substances to the microbial food web. Irrespective of phylum or genus levels (Figures 5, 6), the Spearman’s correlations reveal that OC and pH are two major environmental factors affecting the bacterial community composition in various habitats of the Fildes region. Previous studies have reported that OC and/or pH are common environmental factors influencing bacterial community variation in soil, freshwater lake, and cove of the Fildes region (Wang et al., 2015; Zhang Y. et al., 2018; Nopnakorn et al., 2023; Zhang C. et al., 2024) in the Fildes region. In addition, total organic matter has been found to contribute to the bacterial community structure in intertidal sediments of Fildes Peninsula (Wang et al., 2016).

At the genus level, *Aquibacter*, *Lutibacter*, *Lutimonas*, *Maribacter*, *Marinifilum*, *Maritimimonas*, and *Ulvibacter* within the *Bacteroidota*, *Colwellia*, *Granulosicoccus*, *Psychrobacter*, *Psychromonas*, and *Woeseia* within the *Gammaproteobacteria*, SEEP-SRB4 and *Desulforhopalus* within the *Desulfobacterota*, and *Fusibacter* within the *Firmicutes* were positively correlated with both OC and pH. These genera were exclusively detected or abundant in marine samples with relatively high nutrient content and alkaline pH. They have been frequently observed in marine sediments (Purdy et al., 2003; Zeng et al., 2017; Hicks et al., 2018; Müller et al., 2018; Peoples et al., 2018; Fang et al., 2019; Rizzo et al., 2019; Hoffmann et al., 2020; Brioukhanov et al., 2023; Kachiprath et al., 2024). Simultaneously, *Gaiella*, *Marmoricola*, *Nocardioides*, *Oryzihumus*, and CL500-29_marine_group within the *Actinobacteriota*, RB41 and g__norank_f__Vicinamibacteraceae within the *Acidobacteriota*, *Polaromonas*, Ellin6067, and g__norank_f__A21b within the *Burkholderiales* of *Gammaproteobacteria*, C0119, Gitt-GS-136, KD4-96, and P2-11E-related groups within the *Chloroflexi*, and *Deinococcus* within the *Deinococcota* were negatively correlated with OC or pH. Higher proportions of the sequences related to *Actinobacteriota, Acidobacteriota, Burkholderiales*, and *Chloroflexi* were generally observed in pristine soils (low OC and weakly acidic pH) than in marine sediments (high OC and alkaline pH). *Deinococcota*-related sequences were absent from marine sediments. Members of these bacterial groups are usually reported in terrestrial environments, including Antarctic soils (Shravage et al., 2007; Babalola et al., 2009; McIlroy et al., 2015; Lanzén et al., 2016; Frey et al., 2021; Miao et al., 2021; Zhang et al., 2021; Jurelevicius et al., 2022; Li et al., 2022; Díaz et al., 2023; Wei et al., 2023; Wang et al., 2024), suggesting that they can adapt to cold and oligotrophic soils and play a role in nutrient turnover in the harsh habitats.

Moisture is one of important factors determining soil bacterial community structure in the Antarctic (Ganzert et al., 2011; Stomeo et al., 2012; Kim et al., 2020; Zhang E. et al., 2024). However, it shows no significant correlation with soil bacterial community composition in the Fildes Region (Wang et al., 2015). This may be due to the maritime climate of the Fildes region characterized by constant high atmospheric humidity.^12^ Salinity is another environmental variable that is significant correlated with Antarctic soil microbial diversity (George et al., 2021). In addition, the ammonia-oxidizing archaea and bacteria community composition has been reported to shift across the coastal soil-interface-sediment gradient with salinity identified as one of major environmental drivers (Zhang L. M. et al., 2018). The increase in moisture may alter conditions for soil microbial communities by diluting and mobilizing salts (Van Horn et al., 2014). Therefore, the influence of moisture and salinity on microbial community composition and function in different habitats in the Fildes region should be considered to perform in the future.

### 4.3 Functional potential and indicator species

Shotgun metagenomic analysis revealed that bacteria predominated in all four habitats (Table 4), indicating that bacteria play important roles in the ecosystems of the Fildes region. Consistent with the 16S rRNA sequencing results, *Proteobacteria*, *Actinobacteria*, and *Bacteroidetes* were found to be dominant across all samples. *Proteobacteria* (e.g., *Alpha-, Beta-*, and *Gammaproteobacteria*), *Actinobacteria* (e.g., *Actinobacteria*), and *Bacteroidetes* (e.g., *Flavobacteriia*) dominated in bacteria involved in carbon, nitrogen, and sulfur metabolism, supporting the fact that bacteria are an essential part of the food web in polar ecosystems, contributing to nutrient cycling and energy flow (Cavicchioli, 2015; Cao et al., 2020; Cowan et al., 2014).

Simultaneously, similar to 16S rRNA sequencing data, differences in microbial assemblages were observed between the marine and terrestrial samples (Supplementary Table S1). In addition, KEGG annotation results (Figure 9A) exhibited differences in functional properties between terrestrial and marine ecosystems. This implies the influence of harsh and changing terrestrial environments (e.g., low temperature, drought, and high UV radiation) on microorganisms via various adaptation mechanisms, such as synthesis of cold-adapted proteins (e.g., psychrophilic enzymes, cold shock proteins, and ice-binding proteins), compatible solutes (e.g., glycerol and trehalose), and photolyases (Li C. et al., 2015; He et al., 2019; Guo et al., 2021) and regulation of cell membrane fluidity (e.g., branched-chain and polyunsaturated fatty acids) (Shen et al., 2021; Ramasamy et al., 2023). As described above, in this study, genes related to repair of DNA damage and salt and osmotic stress tolerance were found to be more abundant in pristine soils, while genes associated with cold adaptation were in higher abundance in marine sediments. Osmotic stress-tolerant bacteria are helpful for plant growth under drought stress conditions (Patel et al., 2022). Soil microorganisms have the capacity to synthesize glycine betaine (*betB*; Scholz et al., 2016), glutamate (*gltB*; Csonka et al., 1994), and trehalose (*treS*; Kimura et al., 2014) as osmoprotectors. In addition, Na^+^-H^+^ antiporter gene *nhaA* and osmotically inducible protein C gene *osmC* can be important for drought and salt tolerance in soil bacteria (Guo et al., 2020; Serrano, 1996; Shin et al., 2004). The gene encoding single-stranded DNA-binding protein (SSB) was more abundant in marine sediments. Previous study suggests that SSBs can provide a useful system for exploring the adaptation of protein-protein and protein-DNA interactions at low temperature and high pressure (Chilukuri and Bartlett, 1997).

Furthermore, differences in ecological functions of microbes in the four different habitats were revealed in this study. For example, compared to microbes in pristine soils playing a more important role in phosphorus solubilization, microbes in ornithogenic soil performed a more substantial function in denitrification, microbes in intertidal sediments played a more significant role in thiosulfate oxidation, and microbes in marine sediments were more closely involved in sulfate reduction (Figure 9B).

Methane is one of the most important greenhouse gases associated with global climate change. The AP and nearby islands have experienced a marked warming trend in the past 50 years, potentially resulting in higher methane emissions from this area (Roldán et al., 2022). However, methane emissions from Livingston island soil were found lacking and can be explained by complete oxidation of methane by methane-oxidizing bacteria (MOB; Ganzert et al., 2011). MOB are able to use methane for growth, potentially being an indicator of negative average methane flux (Ganzert et al., 2011; Roldán et al., 2022). In this study, consistent with the presence of particulate methane monooxygenase *pmoA* gene (Figure 9B), methane-oxidizing bacteria *Methylotenera* and non-methane-oxidizing bacteria *Methylophilaceae* were more abundant in the terrestrial samples than in the marine samples. Methanotrophs are often associated with specific non-methanotrophic bacteria in environment samples, suggesting a metabolic framework for methane oxidation by communities of different metabolic guilds rather than methanotrophs alone (Hernandez et al., 2015). In contrast, the phylum *Methylomirabilota*, which are known as anaerobic methanotrophs (Roldán et al., 2022), was detected in the terrestrial samples in very small amounts (< 0.1%). Bacterial oxidation can consume > 99% of methane existing beneath the ice sheet in the subglacial lake Whillans in West Antarctica, representing a significant methane sink (Michaud et al., 2017). The study findings will be helpful to answer the question as to whether the ice-free polar regions can act as atmospheric methane sink.

Different from *pmoABC* genes observed in the four investigated habitats, methyl-coenzyme M reductase (MCR) genes *mcrABG* were exclusively detected in the marine sediments. MCR is a key enzyme in (reverse) methanogenesis and is found in anaerobic methanotrophic archaea (Laso-Pérez et al., 2016). Anaerobic oxidation of methane (AOM) by archaea through reverse methanogenesis is a major process through which methane is consumed in marine sediments (Knittel and Boetius, 2009). Similar to a previous report of anaerobic methane-oxidizing archaea coexisting with sulfate-reducing bacteria in marine sediments (Wang et al., 2019; Begmatov et al., 2021), relatively more abundant sulfate reduction-related genes (e.g., *dsrAB* and *sat*) and related bacterial genera (e.g., *Desulfobulbus*, *Desulfoconvexum Desulforhopalus*, and *Desulfuromusa*) were detected in marine sediments in this study. This suggests that archaeal AOM coupling co-exists with bacterial sulfate reduction in the marine sediments of the Fildes region. *Desulfobacterota* (formerly as *Deltaproteobacteria*) are known for their ability to respire sulfate by utilizing protein complexes, such as sulfate adenylyltransferase (Sat), adenylyl sulfate reductase (Apr; most abundant in marine sediments compared to other three habitats), and dissimilatory sulfite reductase (Dsr) (Langwig et al., 2022). *Desulfobacterota-*related sequences were most abundant in the marine sediments than in the other habitats, indicating an important role of bacteria in sulfur cycling in marine sediments of the Fildes region. *Desulfatiglandaceae*, *Desulfobacteraceae*, and *Desulfobulbaceae-*related sequences were exclusively abundant in the marine sediments in this study, consistent with the finding that these bacteria are dominant in sulfate-reducing microorganisms in global marine sediments (Robador et al., 2016). Sulfate reduction dominates in Antarctic marine sediments (Purdy et al., 2003). The sulfur cycling is driven by anaerobic microorganisms performing dissimilatory sulfate reduction, which is coupled to anaerobic oxidation of methane or organic matter and is important for lithotrophy and organic matter mineralization (Jørgensen and Kasten, 2006; Jørgensen et al., 2019; Wurgaft et al., 2019). In addition, AOM is reported to couple to nitrate reduction in archaea (Haroon et al., 2013). Dissimilatory nitrate reduction-related genes (e.g., *napA*) were more abundant in the marine sediments among the four habitats, though it is uncertain if these genes originated from archaea or bacteria. Though the nitrogen-fixing *nifH* gene was found to be most abundant in the marine sediments (Figure 9B), its relative abundance was much lower than genes *napA* and *nosZ* related to denitrification as well as genes *nirB*, *glnA*, and *gltB* involved in L-glutamate synthesis, indicating the minor contribution of nitrogen fixation to the overall nitrogen metabolism in marine sediments. Archaea may play important roles in methane metabolism coupled with sulfur and nitrogen metabolism in Antarctic marine sediments. Therefore, study on diversity and function of archaeal community in marine sediments of the Fildes region should be conducted in the future.

Flavocytochrome *c* sulfide dehydrogenase gene *fccAB* related to sulfide oxidation was more abundant in the marine sediments than the widely distributed (in all habitats) sulfide-quinone oxidoreductase gene *sqr*. Sulfide dehydrogenases are widely distributed in chemolithotrophic sulfur-oxidizing bacteria, such as *Thiomicrospiraceae* (Brinkhoff and Muyzer, 1997; Lü et al., 2017). In this study, *Thiomicrospiraceae*-related sequences were exclusively detected in very low abundance (< 0.05%) in the marine sediments. The relative abundance of *sqr* was found to be at least 10-fold greater than that of *fccAB* in the Fildes region. In contrast, thiosulfate-oxidizing genes (e.g., *soxXYABC*) were most abundant in the intertidal sediments than in the other habitats. Thiosulfate is a key intermediate in the oxidation of hydrogen sulfide to sulfate in oxygenated surface sediments, serving as a direct product for 68–78% of sulfide oxidation (Jørgensen, 1990; Jørgensen et al., 2019). Compared to *soxC* gene being absent from many green and purple sulfur bacteria (Dahl, 2017), *soxB* gene is present in many *Roseobacter* strains (Luo and Moran, 2014). *Roseobacter* clade bacteria within the *Alphaproteobacteria* are abundant in marine bacterioplankton worldwide and dominant in intertidal sediments, suggesting their ecological role in sulfur oxidation in oxic and suboxic sediment layers (Lenk et al., 2012). *Rhodobacteraceae*-related sequences were dominant (4.1%) in intertidal sediments of this study. The study findings reveal differences in bacterial diversity and metabolic pathways involved in sulfur oxidation between the intertidal and marine sediments in the Fildes region.

With much higher OC content (Table 3), marine sediments contained more abundant genes responsible for carbohydrate catabolism, such as glycoside hydrolases (37.8%) and polysaccharide lyases (3.6%), than terrestrial samples (31.2 and 1.6%, respectively). Microbial functional genes encoding enzymes involved in the degradation of amylum (*amyA*) and hemicellulose (*abfA*) were abundant in the marine sediments (Figure 9B), and chitinase gene (*chiA*) was detected in the marine sediments only. Polysaccharides (e.g., amylum, hemicellulose, and chitin) are produced by plants, algae, diatoms, and crustaceans, which can be deposited in sediments and provide the majority of OC for benthic communities. Sediment microbial communities usually play a critical role in carbon mineralization and cycling of essential nutrients, such as nitrogen and sulfur (Garber et al., 2021).

Soil available phosphorus is essential for plant growth and productivity. The *gcd* gene—encoding the membrane-bound quinoprotein glucose dehydrogenase that governs inorganic phosphate solubilization accompanied by gluconic acid formation—is a determinant predictor of soil available phosphorus and a biomarker for soil phosphorus cycling (Rodríguez et al., 2006; Liang J. L. et al., 2020). The *gcd* genes were abundant in the pristine soils, indicating an ecological role of bacteria in Antarctic soil phosphorus cycling. Soil bacteria harboring *gcd* genes are distributed in *Bacteroidia* (e.g., *Chitinophagales* and *Cytophagales*)*, Alphaproteobacteria* (e.g., *Sphingomonadales*), *Gammaproteobacteria* (e.g., *Xanthomonadales*), *Gemmatimonadetes* (e.g., *Gemmatimonadales*), and *Vicinamibacteria* (e.g., *Vicinamibacterales*) (Wu et al., 2022). In this study, sequences affiliated with *Chitinophagales* (e.g., *Chitinophagaceae*), *Cytophagales* (e.g., *Hymenobacteraceae* and *Spirosomaceae*), *Sphingomonadales* (e.g., *Sphingomonadaceae*), *Xanthomonadales* (e.g., *Rhodanobacteraceae*), *Gemmatimonadales* (e.g., *Gemmatimonadaceae*), and *Vicinamibacterales* (e.g., *Vicinamibacteraceae*) were frequently detected in the pristine soils. However, further studies on the diversity of *gcd*-containing bacteria in Antarctic soils should be carried out. Phosphorus solubilization from insoluble sources by phosphorus-solubilizing bacteria can be mediated by acidification, which is related to gluconic acid production (Wei et al., 2018; Ribeiro et al., 2020). The released soluble phosphorus and organic acid decrease soil pH. It is consistent with the finding of slightly acidic soils in this study (Table 3). The results showed that *Gemmatimonadetes* (*r*^2^ = –0.622, *p* = 0.031) and *Vicinamibacteria* (*r*^2^ = –0.580, *p* = 0.048) were negatively correlated with environmental pH.

Among the carbon fixation pathways, reductive citrate cycle (rTCA), reductive acetyl CoA (Wood-Ljungdahl; WL) pathway, 3-hydroxypropionate (3HP) pathway, 3-hydroxypropionate/4-hydroxybutyrate (3HP/4HB) pathway, and dicarboxylate/4-hydroxybutyrate (DC/4HB) cycle are used predominantly by chemolithoautotrophic bacteria and archaea (Jiang et al., 2022). Abundant carbon fixation genes related to 3HP (e.g., *accABCD*) were detected across all samples, suggesting that 3HP is one of the major carbon fixation pathways in various habitats of Fildes region. The 3HP has been observed in marine sediments (Jiang et al., 2022) and Antarctic soil (Lezcano et al., 2019). Enzymes for 3HP are detected not only in members of *Chloroflexota* (e.g., photosynthetic green non-sulfur bacterium *Chloroflexus*) but also in *Actinobacteria* and *Proteobacteria* (including *Alpha*- and *Gammaproteobacteria*) (Garritano et al., 2022). Chemolithoautotrophic microbes in marine sediments can fix inorganic carbon independent of light (Middelburg, 2011). Genes related to reductive pentose phosphate cycle (Calvin; e.g., *rbcL*) were much more abundant than those related to rTCA (e.g., *aclAB*) in all the samples, suggesting that Calvin is another carbon fixation pathway more frequently adopted by microorganisms in the Fildes region. Ribulose-bisphosphate carboxylase (RuBisCO) is a key enzyme responsible for CO_2_ fixation in Calvin cycle, which is the most common carbon fixation pathway for phototrophs and chemotrophs (Niederberger et al., 2015). The large chain of RuBisCO is encoded by the *rbcL* gene in cyanobacteria and algae. The *rbcL* sequences detected in marine sediments could be partly derived from cyanobacterial and algal detritus from upper waters (Garber et al., 2021). The marine sediments harbored much more abundant WL pathway genes (e.g., *acsABCD*) and 3HP/4HB pathway genes (e.g., *abfD*) than the other three habitats. The WL pathway—requiring strictly anaerobic conditions—has been observed in methanogenic archaea and some bacteria, such as *Desulfobacterium* (Probst et al., 2014). *Desulfobacterium* was exclusively detected in the marine sediments of this study. The 3HP/4HB cycle and 3HP bi-cycle can function under aerobic conditions (Jiang et al., 2022) and has been identified in bacteria (e.g., *Metallosphaera* and *Sulfolobales*) and archaea (Liang B. et al., 2020). Sequences related to *Metallosphaera* and *Sulfolobales* were observed in the four habitats of this study. Collectively, the study findings indicate more diverse carbon fixation pathways existing in the marine sediment habitats than in the terrestrial habitats of the Fildes region. Marine sediments are the largest carbon sink on earth, and nearly half of dark carbon fixation in the oceans occurs in coastal sediments (Dyksma et al., 2016). *Gammaproteobacteria* has been found to drive important parts of marine carbon and sulfur cycles via carbon fixation and sulfur oxidation, respectively, in coastal sediments (Dyksma et al., 2016). In this study, *Gammaproteobacteria* (including *Luminiphilus* and *Thiomicrospira*) accounted for 22.9 and 27.1% of bacteria involved in carbon and sulfur metabolism in the marine sediments, respectively. In the pristine soils, these bacteria proportion decreased to 3.6 and 4.6%, respectively. Contrary to previous reports that Calvin, rTCA, and WL pathways are main carbon fixation pathways present in cold marine sediments (Garber et al., 2021; Jiang et al., 2022), the 3HP, Calvin, WL, and 3HP/4HB were found to be main carbon fixation pathways in the marine sediments of the Fildes region.

Carbon fixation is a critical process in oligotrophic Antarctic soils and may represent the major source of carbon in these arid environments (Niederberger et al., 2015). Endemic biota encompassing members of the cyanobacteria, actinobacteria, proteobacteria, and algae can make a large contribution to soil OC and replenish carbon stocks in Antarctic soils most likely via Calvin pathway (Chan et al., 2013; Niederberger et al., 2015). The Calvin cycle can play a vital role in soil CO_2_ fixation under low mean annual precipitation (MAP) conditions, whereas the rTCA and 3-HP pathways are important under high MAP conditions (Huang et al., 2022). In the pristine soils of the Fildes region, 3HP, Calvin, and 3HP/4HB pathways played an important role in carbon fixation.

Consistent with previous studies (Barbosa et al., 2016; Guo et al., 2018; Prekrasna-Kviatkovska et al., 2024), relatively higher abundance of the genera *Gottschalkia*, *Tissierella*, and *Proteiniclasticum* within the *Clostridia* associated with birds’ guts was found in the ornithogenic soil than in the other habitats, supporting the direct effect of microbe loading to ornithogenic soils by penguin feces. In addition to direct loading of gastrointestinal microbiota to soil, penguin feces can change soil geochemistry, causing increase in nitrogen and phosphorus compounds, indirectly affecting microbiome composition and function in ornithogenic soils (Prekrasna-Kviatkovska et al., 2024). Much higher contents of ammonium and phosphate were observed in the ornithogenic soil than in the other habitats (Table 3). Additionally, differences in bacterial community compositions were detected between ornithogenic soil and pristine soils (Figure 2). As described above, absent from the marine sediments, *Rhodanobacter* was the most abundant genus in the ornithogenic soil but rare in the pristine soils (0.2%). *Rhodanobacter* may be an essential clade involved in denitrification in acidic soils with high emission of N_2_O (van-den-Heuvel et al., 2010). The genomes of publicly available *Rhodanobacter* isolates contain nearly complete gene set for denitrification pathways, including nitrate reductase gene (e.g., *narGHI*) and nitrite reductase gene (e.g., *nirK*) (Kostka et al., 2012; Prekrasna-Kviatkovska et al., 2024). In this study, more abundant genes (e.g., nitrate reductase *NarG*, nitrite reductase *NirK*, nitric oxide reductase *NorB*, and nitrous oxide reductase *NosZ*) associated with aerobic denitrification pathways were observed in the ornithogenic soil. This suggests an important role of microbial denitrification in the ornithogenic soil than in the other habitats. High relative abundance of denitrification genes (i.e., *nirK* and *nosZ*) and denitrifying bacteria related to *Rhodanobacter* has been observed in marine bird-impacted soils (Prekrasna-Kviatkovska et al., 2024). Collectively, penguin feces supplementation not only affects the bacterial community composition in the ornithogenic soil but also influences their function. The ornithogenic soil can be characterized by indicator species of the genus *Rhodanobacter*, which is predicted to be more involved in nitrogen cycling and used to help predict the response of bacterial communities to environmental changes.

In this study, inconsistence of bacterial taxonomic position was observed between the Silva SSU database for 16S rRNA sequencing data and the NCBI NR database for metagenomic shotgun sequencing data. For example, the *Burkholderiales* (e.g., *Comamonadaceae*) and *Desulfobacterales* (e.g., *Desulfoconvexum*) were classified into *Gammaproteobacteria* and *Desulfobacteria*, respectively, according to the Silva database but they were clustered within the *Beta*- and *Deltaproteobacteria*, respectively, based on NR and LPSN databases (list of prokaryotic names with standing in nomenclature).^13^ Such inconsistency leads to different results showing that *Beta-* and *Deltaproteobacteria* were absent from bacterial communities based on 16S rRNA sequencing data but were dominant in bacterial communities and important for carbon, nitrogen, and sulfur cycling based on metagenomic shotgun sequencing data (Figure 8). Additionally, inconsistency in bacterial taxonomic positions in the microbial community in cold seeps has been observed between the Silva database and genome taxonomy database (Yang et al., 2020).^14^ Therefore, attention should be paid to the reference databases chosen for taxonomic classification.

## 5 Conclusion

This is the first investigation report on the composition and function of bacterial communities in various habitats of the Antarctic Fildes region using a combination of 16S rRNA gene sequencing and metagenomics. Bacterial communities exhibited clear differences in diversity and composition between terrestrial (i.e., pristine and penguin ornithogenic soils) and marine ecosystems (i.e., marine and intertidal sediments). Meanwhile, these habitats harbored their own bacterial groups showing unique characteristics. Overall, microbiota in the terrestrial ecosystem showed relatively more diverse metabolic pathways than in the marine ecosystem. OC and pH were two major environmental factors influencing the bacterial community compositions. Additionally, differences in bacterial functions were observed among the four habitats due to their unique environmental conditions. Penguin feces supplementation could affect the bacterial community composition and function in soils. *Proteobacteria*, *Actinobacteria*, and *Bacteroidetes* were dominant in bacterial communities and played important roles in carbon, nitrogen, and sulfur cycling. The potential keystone taxa (e.g., biomarkers) associated with biogeochemical cycles in different habitats were identified and could further be utilized as indicator species for environmental conditions, such as with *Gemmatimonas*, *Rhodanobacter*, *Roseobacter*, and *Desulfobacteraceae* being involved in anoxygenic photosynthesis and phosphorus acquisition in the pristine soils, denitrification in the ornithogenic soil, thiosulfate oxidation in the intertidal sediments, and sulfate reduction in the marine sediments, respectively. The study findings will be helpful to improve our understanding of composition and function of bacterial communities in various habitats and the response of bacterial communities to the current rapid warming in maritime Antarctic regions. Simultaneously, despite their small proportion in environmental metagenome, archaeal community composition and function should be paid attention in the future.

## Data Availability

The data of 16S rRNA gene high-throughput sequencing and shotgun metagenomic sequencing were deposited to NCBI Sequence Read Archive database under accession numbers PRJNA1173308 and PRJNA1175842, respectively.

## Footnotes

1
http://www.uba.de/uba-info-medien-e/4424.html

2
www.illumina.com

3
https://drive5.com/usearch

4
https://github.com/jstjohn/SeqPrep

5
https://github.com/najoshi/sickle

6
http://metagene.cb.k.u-tokyo.ac.jp/

7
http://www.ncbi.nlm.nih.gov/Taxonomy/taxonomyhome.html/index.cgi?chapter=tgencodes#SG1

8
http://www.bioinformatics.org/cd-hit

9
http://soap.genomics.org.cn

10
https://cran.r-project.org/src/contrib/Archive/vegan/

11
https://cloud.majorbio.com

12
http://www.uba.de/uba-info-medien-e/4424.html

13
https://lpsn.dsmz.de/

14
https://gtdb.ecogenomic.org/

## Supplementary material

Supplementary Figure S1 Rarefaction curves of bacteria in 12 samples collected from the Fildes region. Operational taxonomic units (OTUs) in this analysis were defined at 97% sequence identity.

Supplementary Figure S2 Co-occurrence networks of bacterial communities based on top 50 genera. The size of each node is proportional to the relative abundance.

Supplementary Figure S3 Metabolic pathways detected in terrestrial and marine ecosystems of the Fildes region. Marine ecosystem includes intertidal (i.e., IT1 and IT2) and marine sediments (i.e., Sed1, Sed2, and Sed3); Terrestrial ecosystem includes pristine (i.e., So1, So2, So3, So4, So5, and So6) and ornithogenic soils (Or1).

Supplementary Figure S4 Heatmap showing differences in relative abundance of key function genes associated with aromatic hydrocarbon degradation, cold adaptation, salt and osmotic stress tolerance, and repair of DNA damage in four habitats. So, pristine soil; Or, ornithogenic soil; IT, intertidal sediment; Sed, marine sediment.

Supplementary Figure S5 Venn diagram showing shared and unique bacterial phyla in four habitats of the Fildes region. So, pristine soil; Or, ornithogenic soil; IT, intertidal sediment; Sed, marine sediment.

Supplementary Figure S6 Venn diagram showing shared and unique bacterial OTUs in four habitats of the Fildes region. So, pristine soil; Or, ornithogenic soil; IT, intertidal sediment; Sed, marine sediment.

Supplementary Table S1 Community composition in 12 samples at the phylum level based on metagenomic data.

Supplementary Table S2 Differing KEGG categories in terrestrial and marine ecosystems.
