# Supplementary figures and images for: Bacterial community composition and function in different habitats in Antarctic Fildes region revealed by high-throughput sequencing
Source: Front Microbiol. 2025 Jun 16;16:1524681. doi: 10.3389/fmicb.2025.1524681 (PMC12206884; doi:10.3389/fmicb.2025.1524681)

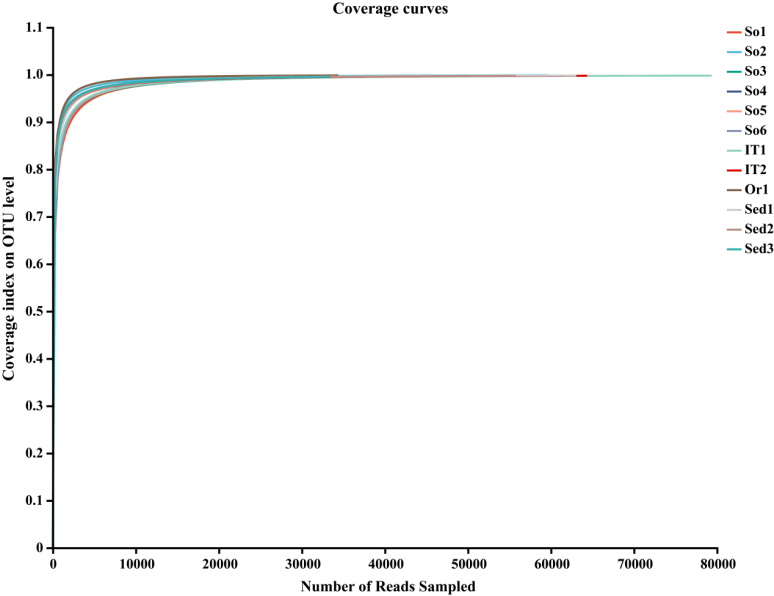

Supplement: Supplementary Figure S1 — Rarefaction curves of bacteria in 12 samples collected from the Fildes region. Operational taxonomic units (OTUs) in this analysis were defined at 97% sequence identity. [file Image_1.png]

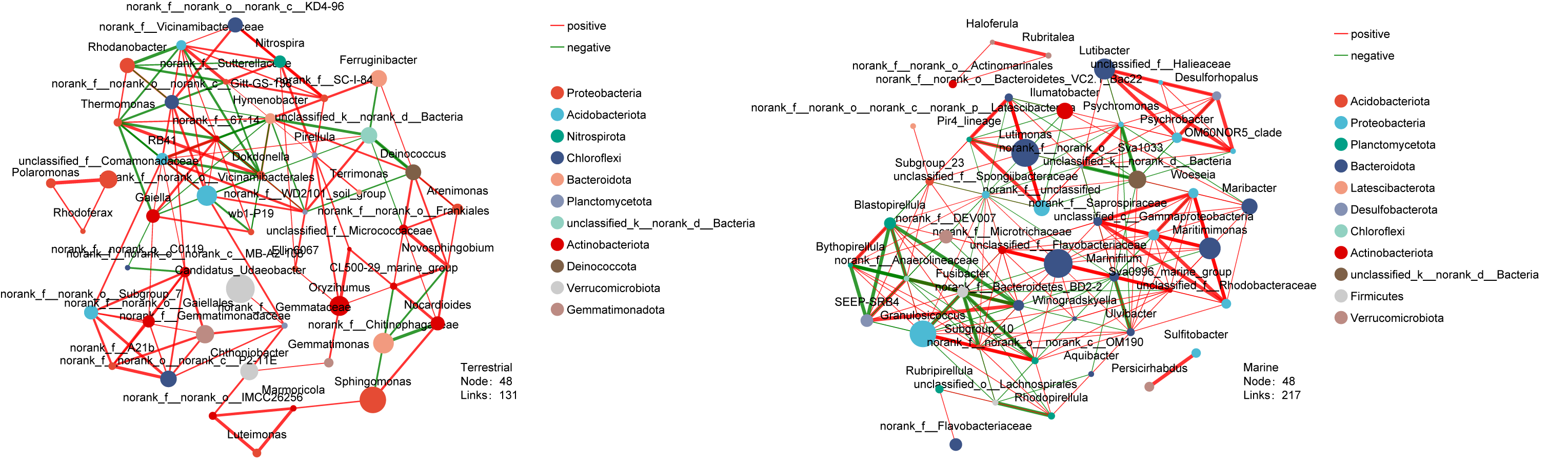

Supplement: Supplementary Figure S2 — Co-occurrence networks of bacterial communities based on top 50 genera. The size of each node is proportional to the relative abundance. [file Image_2.tif]

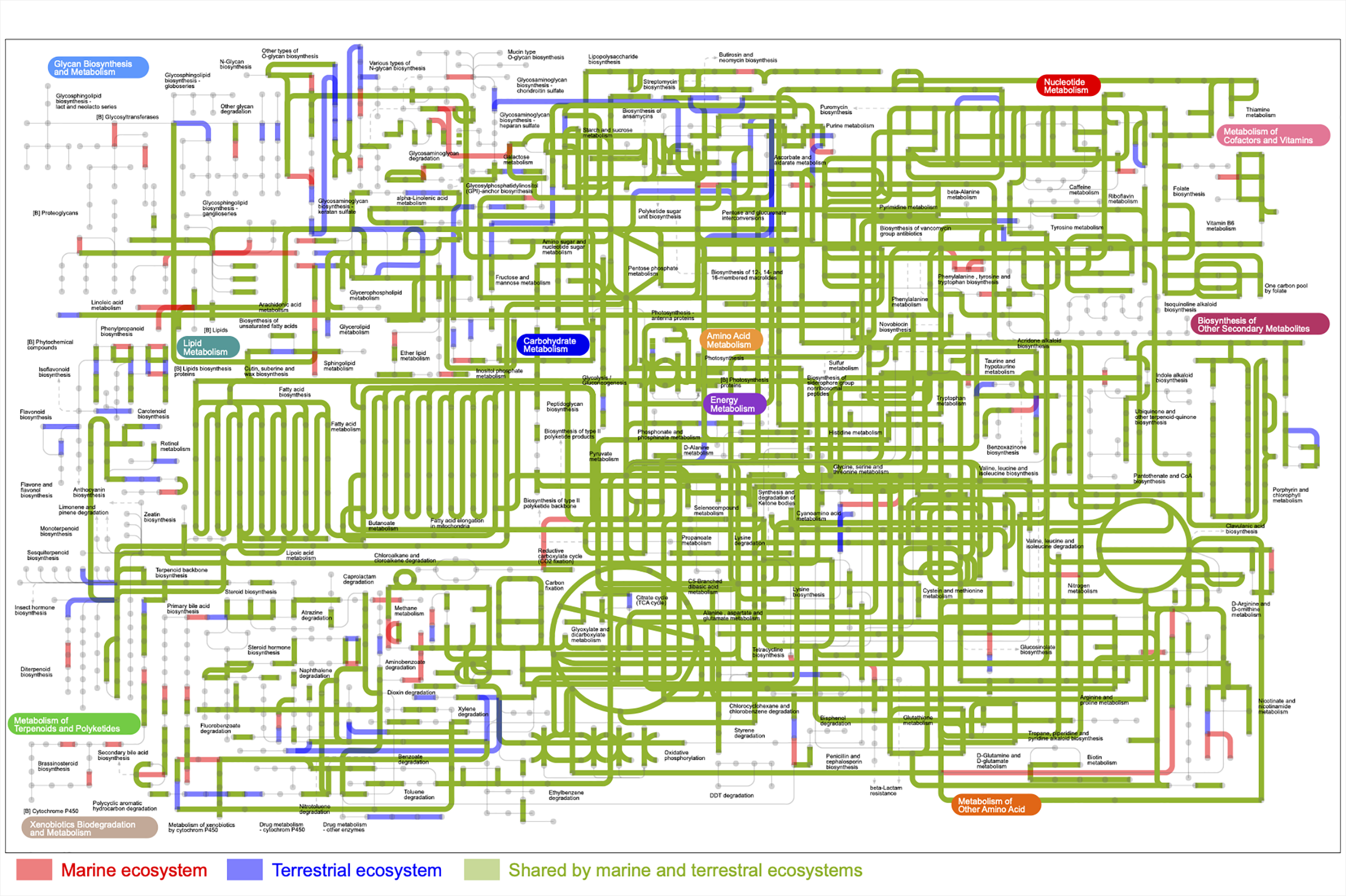

Supplement: Supplementary Figure S3 — Metabolic pathways detected in terrestrial and marine ecosystems of the Fildes region. Marine ecosystem includes intertidal (i.e., IT1 and IT2) and marine sediments (i.e., Sed1, Sed2, and Sed3); Terrestrial ecosystem includes pristine (i.e., So1, So2, So3, So4, So5, and So6) and ornithogenic soils (Or1). [file Image_3.tiff]

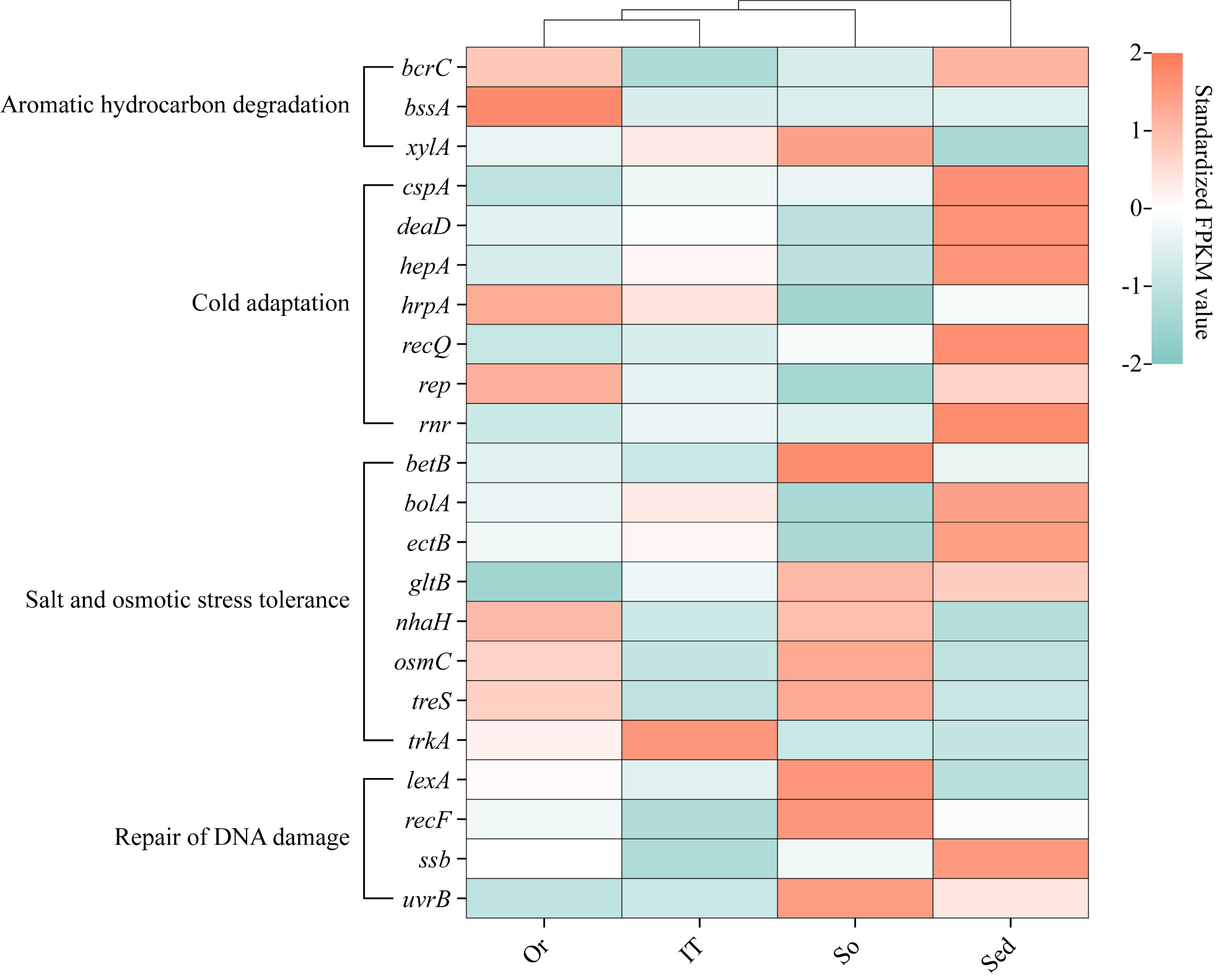

Supplement: Supplementary Figure S4 — Heatmap showing differences in relative abundance of key function genes associated with aromatic hydrocarbon degradation, cold adaptation, salt and osmotic stress tolerance, and repair of DNA damage in four habitats. So, pristine soil; Or, ornithogenic soil; IT, intertidal sediment; Sed, marine sediment. [file Image_4.tif]

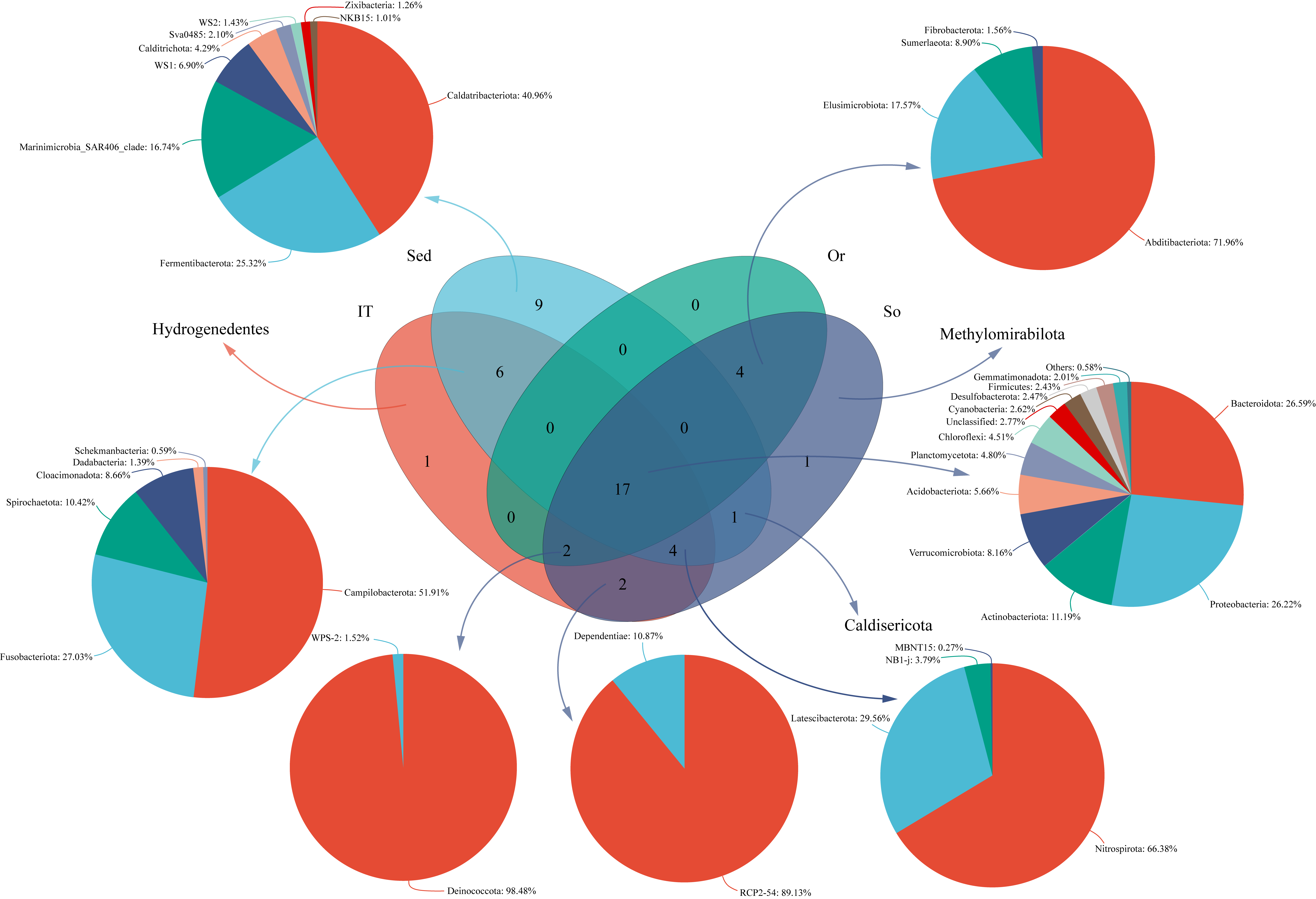

Supplement: Supplementary Figure S5 — Venn diagram showing shared and unique bacterial phyla in four habitats of the Fildes region. So, pristine soil; Or, ornithogenic soil; IT, intertidal sediment; Sed, marine sediment. [file Image_5.png]

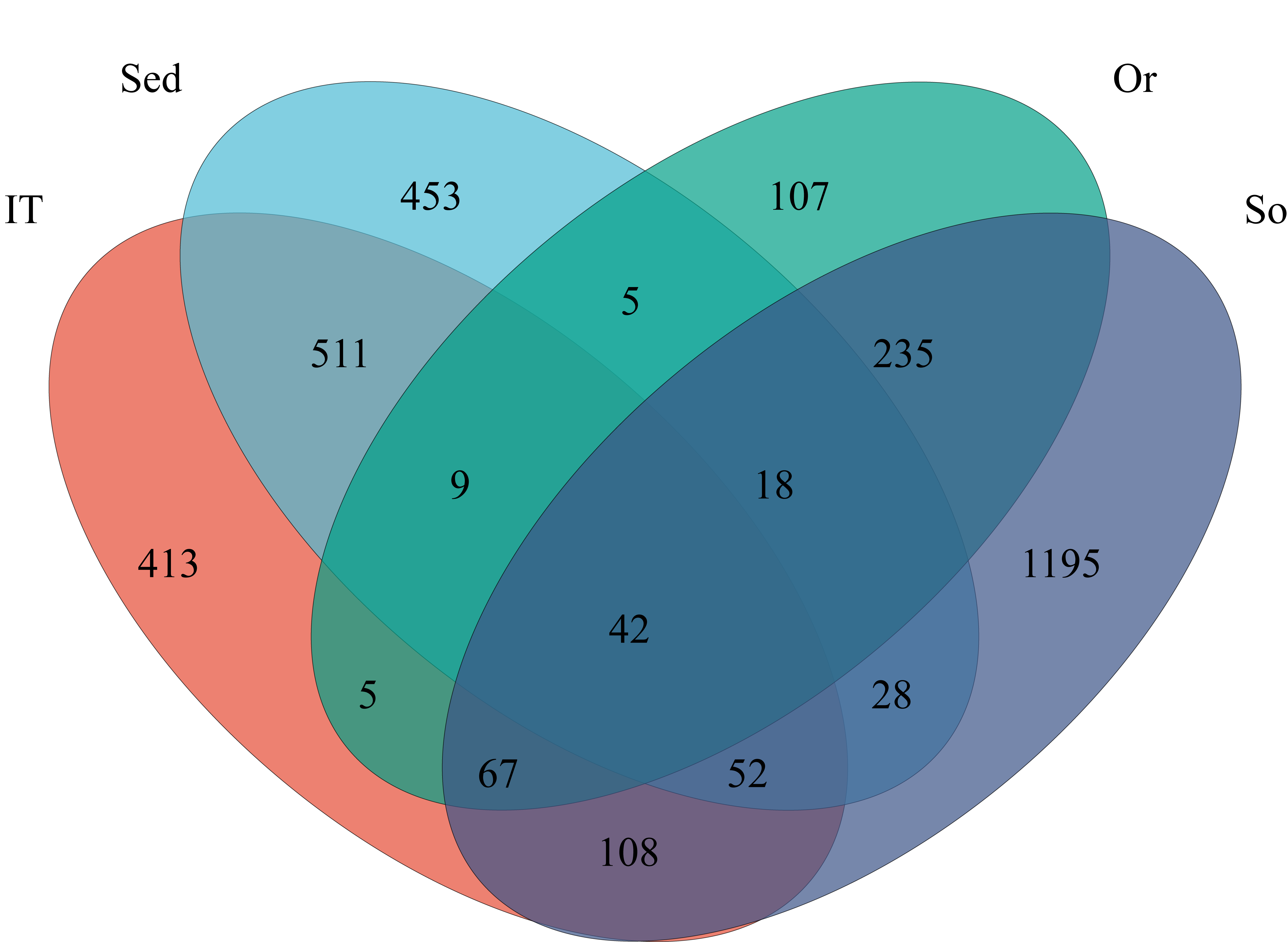

Supplement: Supplementary Figure S6 — Venn diagram showing shared and unique bacterial OTUs in four habitats of the Fildes region. So, pristine soil; Or, ornithogenic soil; IT, intertidal sediment; Sed, marine sediment. [file Image_6.png]
